# Aberrations in temporal dynamics of cognitive processing induced by Parkinson’s disease and Levodopa

**DOI:** 10.1038/s41598-023-47410-3

**Published:** 2023-11-18

**Authors:** Mohammad Mahdi Kiani, Mohammad Hossein Heidari Beni, Hamid Aghajan

**Affiliations:** Department of Electrical Engineering, Sharif University of Technology, Tehran, Iran

**Keywords:** Parkinson's disease, Cognitive ageing

## Abstract

The motor symptoms of Parkinson's disease (PD) have been shown to significantly improve by Levodopa. However, despite the widespread adoption of Levodopa as a standard pharmaceutical drug for the treatment of PD, cognitive impairments linked to PD do not show visible improvement with Levodopa treatment. Furthermore, the neuronal and network mechanisms behind the PD-induced cognitive impairments are not clearly understood. In this work, we aim to explain these cognitive impairments, as well as the ones exacerbated by Levodopa, through examining the differential dynamic patterns of the phase-amplitude coupling (PAC) during cognitive functions. EEG data recorded in an auditory oddball task performed by a cohort consisting of controls and a group of PD patients during both on and off periods of Levodopa treatment were analyzed to derive the temporal dynamics of the PAC across the brain. We observed distinguishing patterns in the PAC dynamics, as an indicator of information binding, which can explain the slower cognitive processing associated with PD in the form of a latency in the PAC peak time. Thus, considering the high-level connections between the hippocampus, the posterior and prefrontal cortices established through the dorsal and ventral striatum acting as a modulatory system, we posit that the primary issue with cognitive impairments of PD, as well as Levodopa’s cognitive deficit side effects, can be attributed to the changes in temporal dynamics of dopamine release influencing the modulatory function of the striatum.

## Introduction

According to statistics, Parkinson's disease (PD) is the second most prevalent neurodegenerative disorder, affecting roughly 2–3% of the population over 65^1^. PD was initially identified as a disease of the motor system. More research, however, revealed that as the disorder progresses, non-motor symptoms—particularly cognitive issues—become more prominent. In advanced stages of PD, dementia may also arise. For a group of Parkinson's patients, non-motor symptoms such as cognitive impairments affecting learning, memory, decision-making, and attention, can be more disabling than movement deficits^2^. Although pharmaceutical treatment or deep brain stimulation have made progress in improving the motor symptoms caused by PD, current treatment approaches are unsuccessful in reducing the cognitive impairments induced by the disease and may even produce undesired side effects^3,4^. Deficit in dopamine (DA) levels accompanied by the loss of dopaminergic neurons in the substantia nigra is known as the primary cause of PD, resulting in dopaminergic impairment in the function of interneurons in the striatum^1^. While many studies suggest links between PD and variations in the spatiotemporal pattern of neural activity^5^, the extent of behavioral and electrophysiological studies on the impacts of PD on the dynamics of dopamine neuromodulation and its effects on the cognitive abilities of the brain remain limited.

Levodopa (L-dopa) is a standard pharmaceutical drug for the treatment of PD, and a significant anti-akinesia effect of Levodopa was observed in human patients of PD^6^. Although clinical studies have revealed considerable improvement in motor symptoms in PD patients due to the replenishment of the missing striatal DA with Levodopa-induced DA, there are no specific conclusions about the pathological manifests of DA deficit in regions involved in PD under a systemic view of the etiology of PD. Hornykiewicz^7^ reported a more extensive deficit of DA in substantia nigra than in striatum, suggesting the striatal DA loss to be the result of the nigral DA loss. This discovery triggered a number of studies to understand the nigra-striatal DA pathways^8–10^.

**Figure 1:**
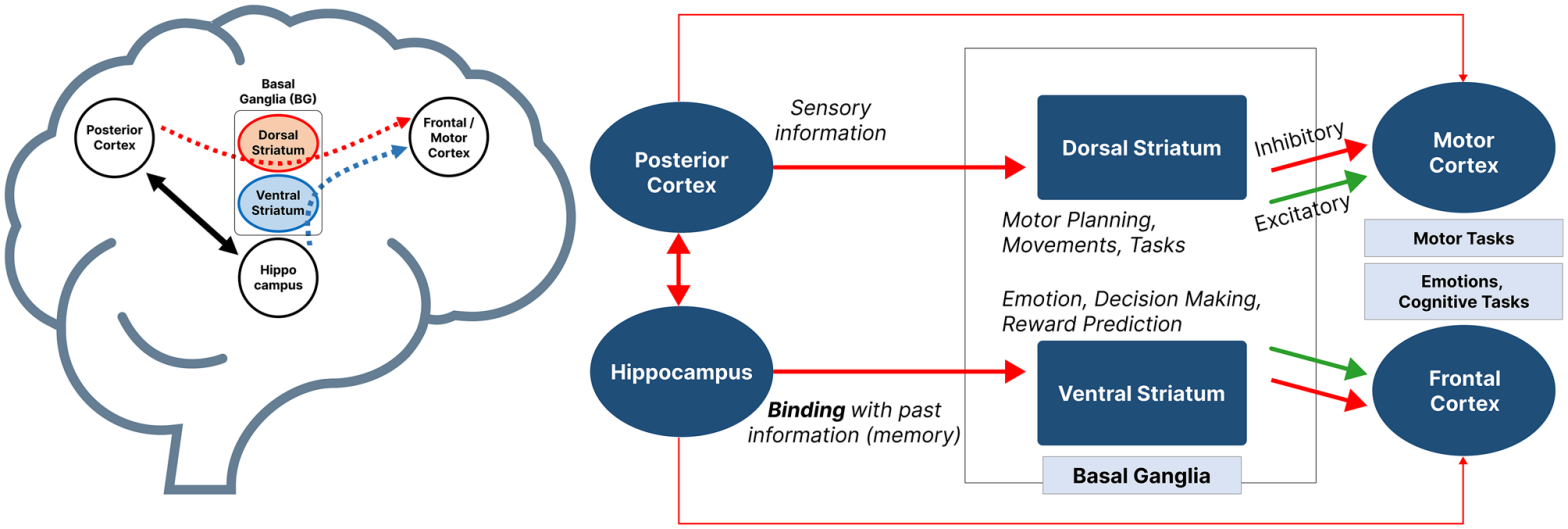
A computational model of basal ganglia. The striatum system is a general structure for handling different kinds of information. Information flows through different paths and is combined with other information resources within the striatal system. The striatal system works with dopamine, which is why PD can cause deficits in the functioning of the striatal system. In the early stages of PD, a deficiency of dopamine is observed in the dorsal striatum, which causes motor disorders, and as the disease progresses, the ventral striatum is also involved, causing cognitive impairments.

To derive a computational view of the structure of the striatum, we can partition the striatum into two regions, ventral striatum (VS) and dorsal striatum (DS)^4^. There are inhibitory and excitatory connections from the striatum to the motor and frontal cortices. Furthermore, there are input connections from the posterior cortex and hippocampus to these two regions of the striatum, see Fig. 1. Considering this network view of the striatum, which is consistent with the computational model suggested in Atallah et al. ^11^, we can point to the essential role that the striatum plays in handling the dynamics of information coming from cortical and subcortical regions^12–17^. Intuitively speaking, each motor movement consists of a sequence of initiating and terminating commands for the movement of muscles through the time. This type of dual action repertoire is also present in conducting cognitive functions such as decision-making, promoting a balance in using sensory information and the past information from memory. The striatum, due to its computational structure shown in Fig. 1, can trigger and conduct these mechanisms giving rise to both motor functions and cognitive functions in the same framework. Through controlling the activation of the inhibitory and excitatory connections form the striatum to the motor and frontal cortices, dopamine plays a critical role in performing motor or cognitive functions, respectively.

The basal ganglia (BG) can be defined as a collection of sub-cortical nuclei in charge of various cognitive tasks, including motor planning^18–20^, reward and sequence learning^21,22^, action selection and decision making^23–25^. The tripartite anatomical model of the basal ganglia developed in^26,27^ suggested pathological reasons for a variety of movement impairments caused by Parkinson's disease, including dyskinesia and akinesia. In clinical terms, akinesia and dyskinesia have been respectively linked to impairment in excitatory and inhibitory connections from the striatum to the motor cortex leading to movement initiation issues and uncontrollable movements. The long-term effect of Levodopa on intensifying dyskinesia has been linked to a mild activation of the inhibitory connection due to redundant DA in the striatum^28^.

**Figure 2:**
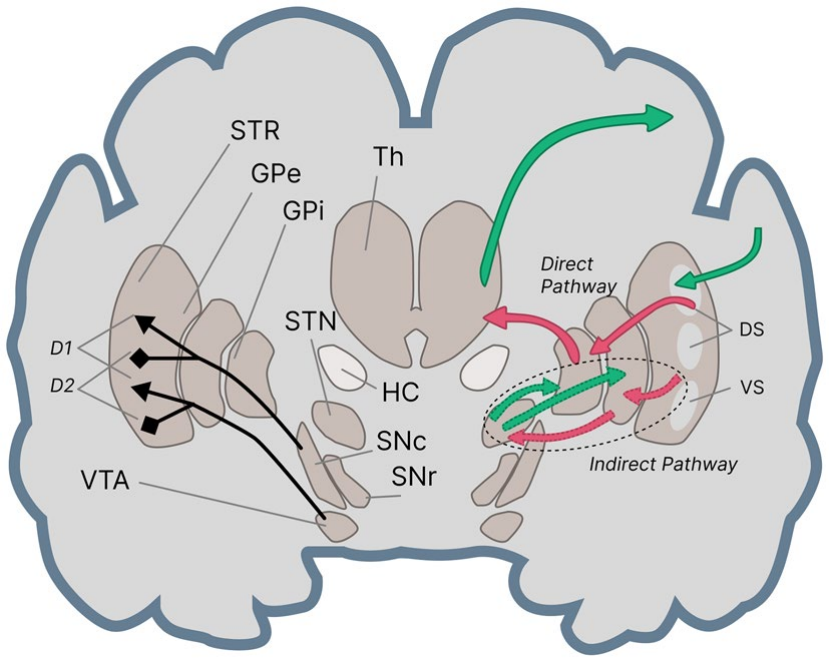
Basal ganglia regions with direct and indirect pathways. STR: Striatum, GPe: Globus Pallidus external, GPi Globus Pallidus internal, STN: SubThalamic Nucleus, Th: Thalamus, HC: Hippocampus, VTA: Ventral Tegmental Area, SNc: Substantia Nigra pars compacta, SNr: Substantia Nigra pars reticular, DS: Dorsal Striatum (including Putamen and Caudate), VS: Ventral Striatum (Nucleus Accumbens (NAc) and Olfactory Tubercle (OT)), D1: Excitatory dopamine receptors, D2: Inhibitory dopamine receptors.

Having said that, we should emphasize that providing a clearer explanation of the dynamics of DA release and its effects on the dynamical activation of the direct pathway's excitatory and inhibitory connections not only provides more insight into the functionality of the basal ganglia in motor planning and cognitive tasks, but can also shed light on the etiology of the dopaminergic neurodegenerative diseases such as PD and explain the movement and cognitive disorders caused by them. The ventral striatum (VS) has been found to play an important role in reward prediction and conditioned behavior^29–31^. Furthermore Tian et al.^32^, showed that a considerable number of input links project from the VS to the midbrain’s dopaminergic neurons, which include the ventral tegmental area (VTA) and the Substantia nigra pars compacta (SNc)^33^, see Fig. 2. In addition, Oettl et al.^17^, demonstrated that phasic dopamine stimulates the formation of a distinct representation for each stimulus in the VS and the olfactory tubercle (OT) nuclei, which then activate dopaminergic neurons differently for each stimulus. Therefore, it has been shown that the VS and midbrain dopaminergic neurons, particularly the VTA, form a loop for establishing reward predictive coding by modulating the dynamics of dopamine release. This loop can also be found in the organization of the basal ganglia, which includes SNc as a component of midbrain dopaminergic neurons, see Fig. 2. Furthermore, the interactions among the nucleus accumbens (NAc) (located in the VS), the hippocampus, prefrontal cortex, and midbrain dopaminergic systems play an important role in goal-directed behavior and reward systems. Moreover, O’Donnell and Grace^15^ and Goto and O’Donnell^34^ showed that activating the hippocampus causes bistable neurons in the NAc to become active and that activating the prefrontal cortex causes spikes in NAc neurons only when they are active. As a result, the hippocampus may be able to control the flow of information from the prefrontal cortex via the VS. Midbrain dopaminergic input into NAc neurons is also important in their state switching and is influenced by hippocampal glutamatergic transmission^35^. Furthermore, the phasic and tonic release of DA regulates the flow of information input from limbic systems, such as the hippocampus, and cerebral cortex, including the frontal cortex, via modulating the activation and inactivation of DA receptors in NAc^16^. This shows the vital role of the VS in the dynamics of information flow between the hippocampus and the prefrontal cortex. In other words, the VS serves the cognitive functions of the brain through combining memory data and novel inputs.

On a large scale, the brain's neuronal network organization facilitates the transfer, integration, and analysis of information, which are fundamental to cognitive activity. A crucial representation of this information processing across brain regions is the Phase-Amplitude Coupling (PAC) of electrical activities. The PAC, a prominent type of cross-band synchronization, arises when the phase of an oscillation at a lower frequency regulates the occurrence of activity at a higher frequency. This neuronal activity pattern has been identified in various mammalian regions, including in the hippocampus, basal ganglia, and neocortex^3^. The PAC has been linked to several cognitive functions, such as working memory, attention, and decision-making^36^.

For attention, a process that filters out extraneous information, studies have shown that the PAC between high gamma (70–250 Hz) and delta (2–5 Hz) or theta (5–8 Hz) bands in a spatial-cueing task can predict reaction time to the task's target stimulus^37^. Furthermore, the role of the PAC in modulating inter-neuron correlations, thereby enhancing the coding scheme for attention direction, has been demonstrated^38^. Building on these studies, other works have associated the PAC between theta-alpha and beta-low gamma bands, and the PAC between the delta band’s phase and high-gamma amplitude, with reaction time during the cueing period and the spatial aspects of the cued spatial attention task, respectively^39^.

In the realm of working memory, and through investigating the phase precession phenomena in hippocampal neurons, Lisman and Buzsáki^40^ introduced a mechanism for encoding sequential information, leading to the association of the PAC between gamma and theta oscillatory activities in the hippocampus. This coding scheme posits that the number of items stored in working memory corresponds to the number of gamma cycles during a theta cycle, defining the pair of frequencies where the PAC is observed during a working memory task. Considering this association, Axmacher et al.^41^ showed that an increase in the number of items stored in working memory leads to a PAC with the lower frequency phase (in theta range) containing a higher number of gamma cycles.

Furthermore, in the realm of auditory cognitive processing, Lizarazu et al.^42^ underscored the significance of the PAC between theta (3–7 Hz) and low gamma (22–35 Hz) frequencies in speech comprehension, extending the effects of the PAC beyond mere sensory processing. In addition, due to the sequential structure of auditory stimuli, temporal patterns of information processing in the brain become more important during auditory processing. For instance, García-Rosales et al.^43^ highlighted the dynamic coupling between the frontal cortex and the auditory cortex in forming the encoding strategy for sounds. In neurodegenerative diseases, the dynamics of functional connectivity between different brain regions can be utilized as a topographical marker for the disease^44^. Specifically, in the context of PD, several studies have explored the impairment in the dynamics of functional connectivity^45,46^ and have associated the severity of the disease to the characteristics of such dynamics^47^. Additionally, many other studies have reported slowing of cognitive processing in PD patients^48–50^. As such, examining the changes in the dynamics of the PAC patterns over time between the patient and control groups can potentially provide further insight into the pathology of these diseases.

Therefore, due to the importance of handling the dynamics of information in cognitive functions such as attention, working memory and auditory cognitive processing, in the current study we investigate the temporal dynamics of the PAC as an indicator of information processing in a task involving attention and working memory^51^. Although the temporal features of EEG signals such as the ERP components P3 and N2^52^, mismatched negativity (MMN)^53^, and delta response^54^ play a critical role in demonstrating discriminative brain activities of PD patients, focusing on the PAC and its underlying information theoretical concepts may lead to a better understanding of the source of cognitive impairments in PD^36^. Furthermore, highlighting the importance of the PAC in cognitive information processing, Bayraktaroğlu et al.^55^ reported its abnormal behavior under an oddball task in Parkinson patients with mild cognitive impairment (MCI). However, the focus of these studies has not been on the temporal patterns of the PAC during cognitive processing. Therefore, noting the results in Gong et al.^56^ suggesting that the PAC calculated between different brain regions better correlates with the severity of motor impairment symptoms, we aim to study the temporal dynamics of the PAC across the scalp to better understand the impairments associated with PD. The latency and amplitude of the MMN and P3 components in oddball experiments have been extensively investigated as markers for healthy brain operation^57–59^. The current study aims to evaluate the latency and amplitude of the PAC^60^ to gain further insight into the dynamics of information processing, and thereby help explain the cognitive deficits caused by imbalances induced in the dopaminergic pathways affected in PD. To this end, we analyze the differences in the temporal behavior of the PAC between PD groups on and off Levodopa with a control group, and develop explanations for the observed differences based on the computational model of the basal ganglia network and the role of dopamine in facilitating cognitive functions in this network. This analysis can provide a foundation for a network-level description of the mechanisms involved in cognitive impairments associated with PD and additionally induced by Levodopa.

## Results

### PAC frequency region selection

To select a differentiating frequency region, we employed statistical analyses on the PAC features. These features were calculated for the low frequency range of 1–20 Hz and high frequency range of 1–80 Hz and averaged into 4 × 4 bins. In order to select a frequency region for the PAC activity which best distinguishes the participant groups, we applied hypothesis testing between each pair of the PD OFF, PD ON, and CTL groups followed by the Benjamini–Hochberg correction for multiple frequency regions tests. As a result, we found significant differences in the frequency range composed of 5–8 Hz for low frequencies and 33–36 Hz for high frequencies between the CTL and the PD ON groups (t-test *p*-value < 0.05), and between the CTL and PD OFF groups (t-test *p*-value < 0.05). Furthermore, there were no noticeable differences between the PAC of the PD ON and PD OFF data groups in these frequency ranges (t-test *p*-value > 0.05). These frequency ranges were selected for further analysis to allow for investigating Parkinson-induced impairments which are not modified by the administration of Levodopa.

### PAC topography

**Figure 3:**
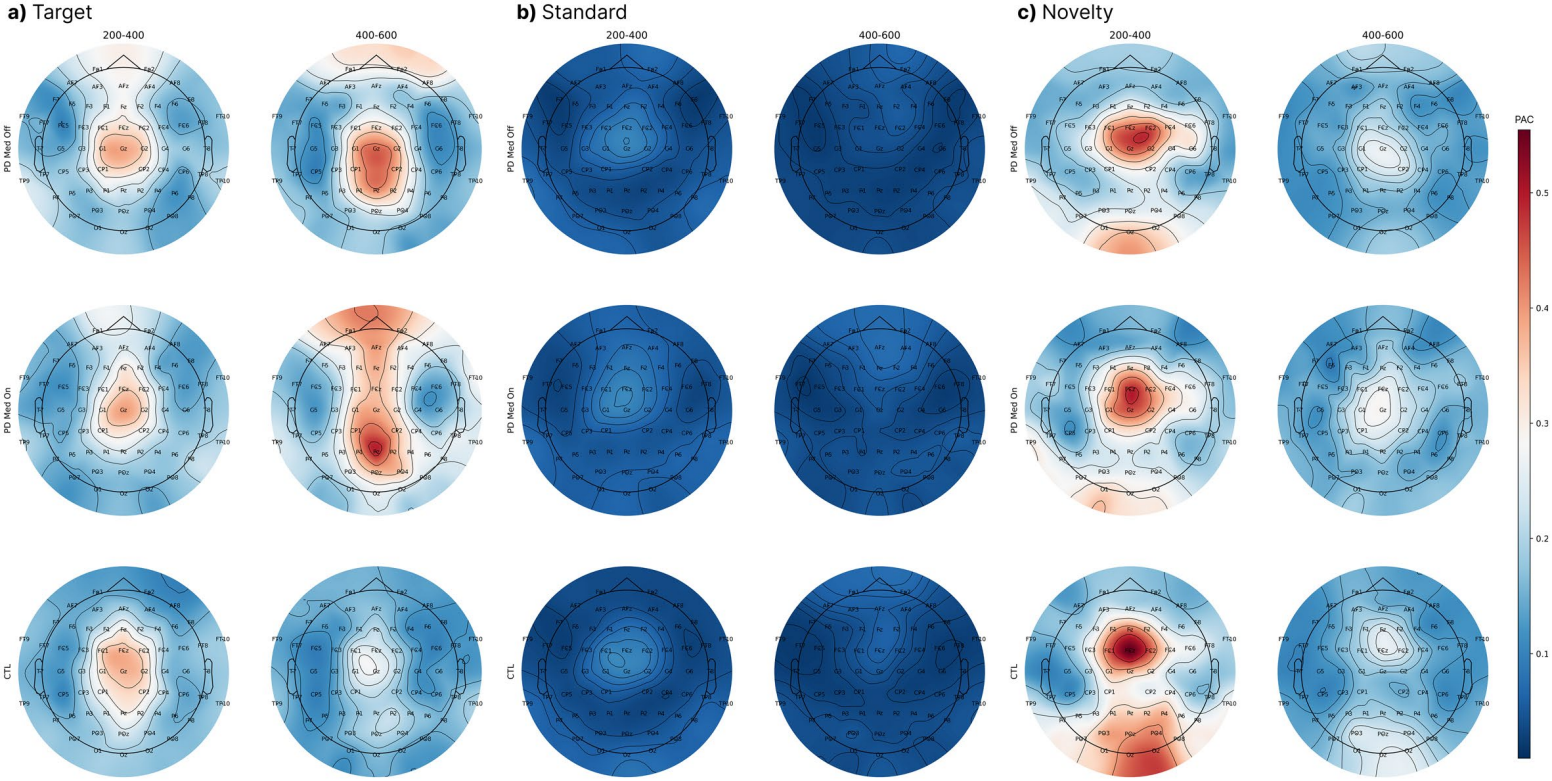
Grand mean values are presented in the topography of the PAC during the time windows of 200–400 ms and 400–600 ms for the stimuli types: (**a**) Target (**b**) Standard (**c**) Novelty. As can be seen, the target PAC activity dynamics differ between both PD groups and the CTL group. However, the dynamics of the PAC response to both novelty and standard stimuli are more comparable between these groups. This indicates that the variations seen for the target stimuli are related to the working memory and cognitive processing functions, which are important in responding to the target stimuli, rather than being related to sensory processing, which is more prominent in processing the novelty stimuli.

In Fig. 3, the topography of the PAC grand mean is displayed for two time intervals: 200–400 ms and 400–600 ms. This is shown for three types of stimuli: target, standard, and novelty. It can be observed that the PAC values for the standard stimulus are consistently lower than those for the target and novelty stimuli across all subject groups and time intervals. To validate the significance of this assessment, hypothesis testing was conducted on each of the 63 channels comparing the PAC activity of the standard stimulus to either of the novelty and target stimuli for each subject group. The highest *p*-value observed across all these paired tests was 0.001, indicating a significant difference in the PAC values between the standard and the two other stimuli types.

In Fig. 3a and c, distinct spatial and temporal patterns can be observed between different groups for PAC values associated with the target and novelty stimuli. For the CTL group, hypothesis testing was conducted to compare the PAC values of the target and novelty stimuli during the 200–400 ms interval in the posterior region, namely for each of the POz, PO3, PO4, PO7, PO8, P3, and P4 channels. Over these channels, the test yielded a maximum *p*-value of paired t-test of 0.03, indicating a significant difference in the PAC activity between the two stimuli types for the CTL group during this time frame.

Additionally, another hypothesis testing was conducted for the CTL group, focusing on the target stimulus within the centro-frontal region—specifically on channels FC3, FC4, F3, F4, and Fz. This test compared PAC values between the 200–400 ms interval and the 400–600 ms interval. A maximum *p*-value of paired t-test of 0.02 was noted, highlighting a significant change in the PAC activity between the earlier and the later time intervals.

For the PD groups, no consistent trend was observed in the PAC values across the centro-frontal region between the specified two time intervals for the target stimulus. On the other hand, a notable decline in the PAC values across the centro-frontal region was observed for the novelty stimulus during those intervals for all subject groups. Hypothesis testing supports this, with a maximum *p*-value of paired t-test of 0.048 indicating the significance of the decline.

For the target stimuli, both the PD ON and PD OFF groups show a notable rise in the PAC values on channel Pz between the mentioned two time intervals. This trend is backed by hypothesis testing, yielding a maximum *p*-value of 0.006 for both groups. In contrast, for the CTL group, no significant increase in the PAC values was observed on channel Pz, as evidenced by a *p*-value of paired t-test of 0.12.

The plots and the results of hypothesis testing reveal differences in both temporal and spatial behavior of the PAC for the novelty and target stimuli between the CTL group and the two PD groups. Notably, for the target stimuli, the PD groups display distinct PAC behavior compared to the CTL group, especially over the centro-frontal region. Building on this observation and supported by t-test evidence, we selected specific channels (Fz, Pz, F3, F4, FC3, FC4) for further analysis to better understand the PAC dynamics in the context of PD. The next section delves into the details of temporal dynamics of the PAC across various brain regions.

### PAC dynamics

**Figure 4:**
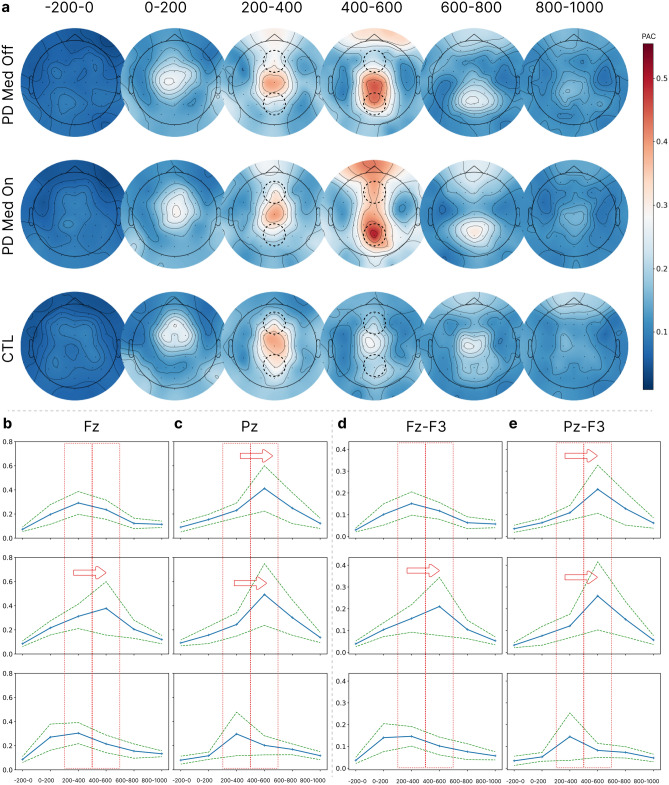
Temporal dynamics of the PAC. (**a**) Topographic maps of the PAC values for the target stimuli for the three subject groups. (**b**, **c**) Temporal dynamics of the PAC for channels Fz and Pz with blue lines for averages over subjects and light margins for ± std/2 over subjects. (**d**, **e**) Temporal dynamics of cross-channel PAC between Fz-F3 and Pz-F3 with blue lines for averages over subjects and light margins for ± std/2 over subjects. The peak of the PAC across the frontal region occurs later for the PD ON group compared to the PD OFF and CTL groups, and can be attributed to the effects of Levodopa usage, while the peak of the PAC across the posterior region occurs later for both PD ON and PD OFF groups compared to the CTL group, and can be attributed to the effects of Parkinson’s disease itself.

Topographical distributions of the PAC values for two time intervals of 200–400 ms and 400–600 ms are shown for the target stimuli in Fig. 3a. The PAC values on the frontal and posterior regions of the scalp demonstrate differences in the dynamics of the PAC between the different subject groups. These differences can be observed in detail for channels Fz and Pz in the PAC temporal dynamics plots of Fig. 4b and c, respectively. As Fig. 4b illustrates, the peak of the PAC on channel Fz occurs in the 400–600 ms interval for the PD ON group, whereas the PAC peak values for the PD OFF and CTL groups on Fz occur in the 200–400 ms interval. In other words, while all groups show an increasing trend in the PAC values for Fz after the stimulus onset, the increasing trend for the PD ON group lasts longer and extends into the 400–600 ms interval in contrast with the PD OFF and CTL groups, which show an increasing trend only until the 200–400 ms interval. The PAC topographic maps of Fig. 4a confirm that such trends also exist for all channels across the frontal region (within the top dotted circles in Fig. 4a). Figure 4c shows the PAC dynamics for channel Pz. For this channel, the peak of the PAC for both PD ON and PD OFF groups occurs in the 400–600 ms interval, demonstrating a marked contrast with the CTL group, for which the peak occurs in the 200–400 ms interval. Similar differences in the dynamics of the PAC values can be seen for other posterior channels (within the bottom dotted circles in Fig. 4a).

### Cross-channel PAC dynamics

Topographic maps of the Fz-F3 and Pz-F3 cross-channel PAC values for the target stimuli are shown in Fig. 4d and e, respectively. The cross-channel PAC dynamics demonstrate similar trends to the single-channel dynamics. Figure 4d shows the temporal PAC dynamics between channels Fz as the amplitude channel and F3 as the phase channel. There is an extended continuation of the increasing trend for the PD ON group compared to the other groups. Similar differences are observed for cross-channel PAC values between Fz as the amplitude channel and F4, FC3, or FC4 as the phase channels (the corresponding plots are included as Fig. S1 in Supplementary Material). Furthermore, in the same way as for the single-channel PAC dynamic for channel Pz, Fig. 4e shows that the peak of the cross-channel PAC dynamics between Pz as the amplitude channel and F3 as the phase channel occurs later for both PD ON and PD OFF groups compared to the CTL group. Similar differences are observed in cross-channel PAC dynamics between Pz as the amplitude channel and F4, FC3, or FC4 as the phase channels (the corresponding plots are included as Fig. S2 in Supplementary Material).

### Hypothesis testing on the PAC peak time intervals

To compare the behavior of PAC dynamics in terms of the time interval in which the PAC peak occurs, the indices of such intervals were determined for each subject. The indices were numbers between 0 and 5 corresponding to the 6 time intervals into which each trial was partitioned. Then, the time interval indices of the PAC peak for the three subject groups were compared, producing *p*-values shown in Table 1. Paired t-test was applied for comparison between the PD ON and PD OFF groups since they consist of the same individuals going through different courses of medication conditions. However, for the comparison of the CTL group with the two PD groups, the independent t-test was applied. As it can be seen, in both Fz PAC and Fz-F3 cross-PAC the time interval of the PAC peak for the PD ON group is different from the CTL (last row) and PD OFF (first row) groups, albeit the *p*-values for comparing the PD ON and PD OFF groups do not satisfy the target significance level of 0.05.

**Table 1:** *P*-values for comparison of the PAC peak time interval index.

*P*-value	Fz-Fz	Fz-F3
PD med off vs PD med on	0.079634	0.054795
PD med off vs CTL	0.213412	0.883191
PD med on vs CTL	0.003487	0.042398

### Comparing PAC dynamics between two block runs

**Figure 5:**
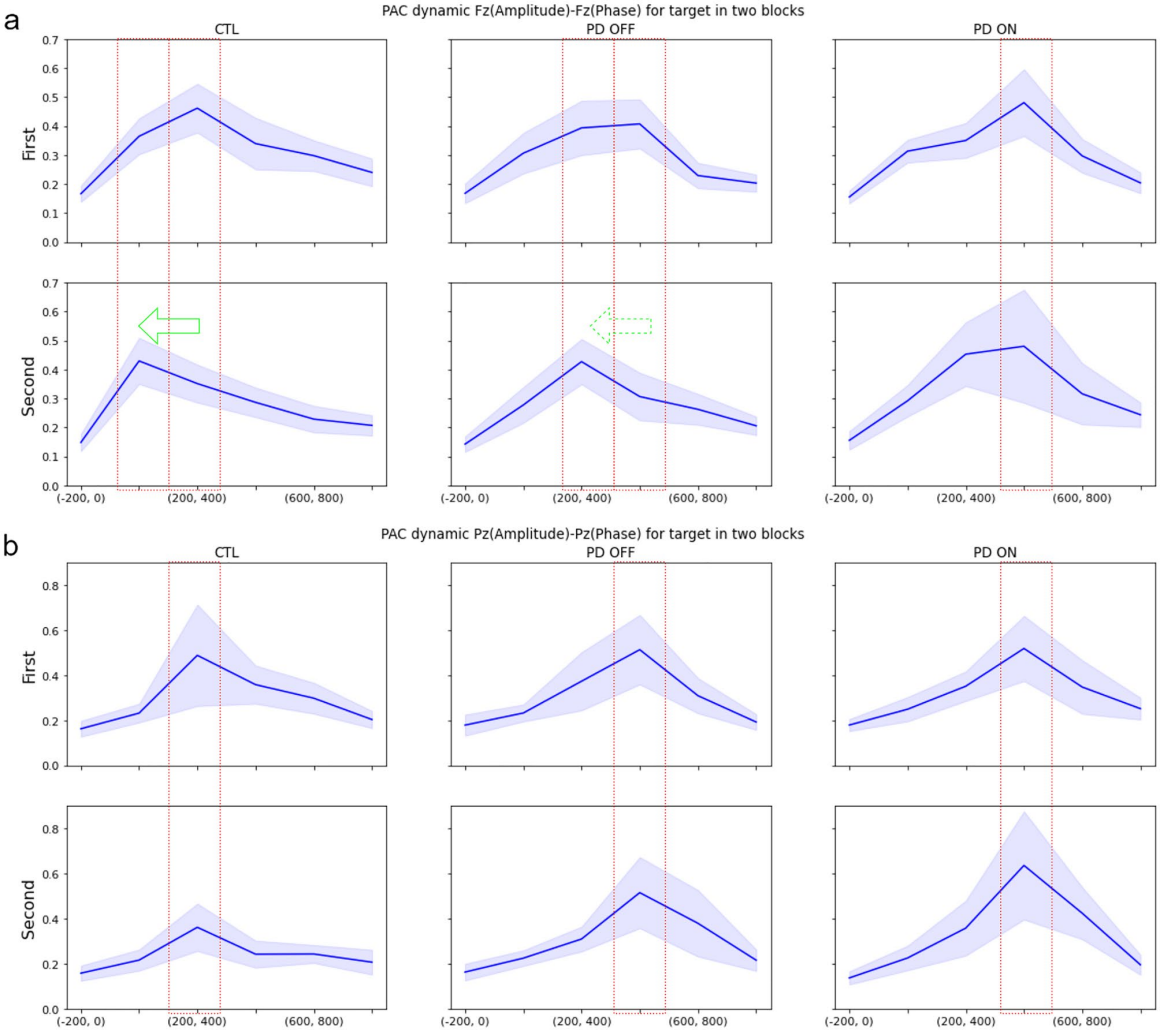
PAC dynamics in two blocks of oddball experiments for channels: (**a**) Fz. (**b**) Pz. Blue lines show averages over subjects and light margins indicate ± std/2 over subjects. It can be seen that the PAC peak in channel Fz occurs earlier in the second block for the CTL and PD OFF groups, but not for the PD ON group. No advancement of the PAC peak occurs for channel Pz.

**Figure 6:**
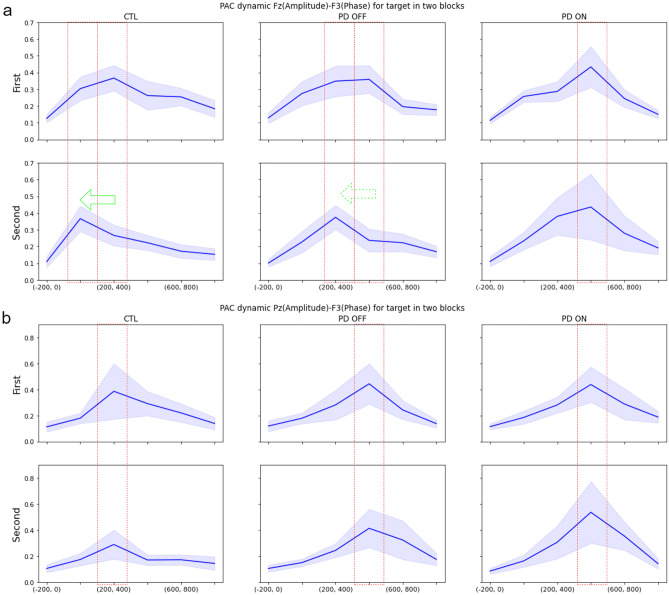
Cross-PAC dynamics in two blocks of oddball experiments for channel pairs: (**a**) Fz (amplitude)-F3 (phase), (**b**) Pz (amplitude)-F3 (phase). Blue lines show averages over subjects and light margins indicate ± std/2 over subjects. It can be seen that the cross-PAC peak in the frontal region (Fz-F3) occurs earlier in the second block for the CTL and PD OFF groups, but not for the PD ON group. No advancement of the peak occurs in the Pz-F3 cross-PAC.

The oddball experiment consisted of two identical blocks of stimuli. Comparing the PAC dynamics for the target stimuli between the two blocks of data reveals that for the CTL group, the peaks of the PAC involving channel Fz in the frontal region, i.e. the Fz PAC (Fig. 5a, left) and the Fz-F3 cross-PAC (Fig. 6a, left), move from the interval of 200–400 ms for the first block to the interval of 0–200 ms for the second block. The PAC dynamics of the PD OFF group (Fig. 5a, middle) demonstrate a similar behavior to the CTL group whereas the PAC dynamics of the PD ON group (Fig. 5a, right) do not show any such advancement for the occurrence of the PAC peak. For the PAC dynamics involving channel Pz in the posterior region (Pz PAC or Pz-F3 cross-PAC) no noticeable advancement of the PAC peak to an earlier time interval is observed for any of the subject groups (Fig. 5b). Similar behavior is observed for other frontal and posterior channel cross-PAC values, which can be seen in Figs. S3, S4, and S5 in Supplementary Material.

## Discussion

Recent studies have implicated deviations in the value of the PAC in Parkinson patients. In particular, Miller et al.^61^ reported that Levodopa reduces excess PAC in the sensorimotor cortex during movement activities and that the resulting reduction in bradykinesia is indeed a motor improvement indicator of Levodopa linked to excess PAC reduction. Furthermore, Gong et al.^56^ discovered an increased level of beta-gamma PAC in Parkinson patients throughout the primary motor cortex, premotor cortex, dorsal lateral prefrontal cortex, and somatosensory cortex. These findings underline the importance of monitoring cross-region coupling of the brain’s oscillations and the critical role that modulating the PAC via Levodopa plays in improving motor symptoms of the disease. Furthermore, Bayraktaroğlu et al.^55^ showed abnormal behavior for the PAC during cognitive activity in PD patients with MCI, and associated it to the impairment in functional connectivity between frontal and parietal regions. Despite these findings, the temporal dynamics of the PAC have not been linked to the effects of PD or Levodopa. Furthermore, there is a lack of knowledge on how the various reported cross-regional PAC behaviors emerge and relate to cognitive and motor impairments. Due to the critical role that basal ganglia circuits, and particularly the striatum, which are most affected by PD play in handling information dynamics^12–17^, a thorough investigation of the temporal dynamics of the PAC, as an indicator of information binding, and the aberrations of these dynamics in PD patients OFF and ON Levodopa was undertaken in the current study. Furthermore, new hypotheses presented in this study on how the PAC dynamics differences between the patient and CTL groups relate to impaired circuits in the striatum can present possible explanations for the cognitive impairments caused by PD and by Levodopa treatment. In addition, since observations of the aberrations in the PAC dynamics in the current study were made on PD patients without MCI diagnosis, our results suggest that the impairments in the fronto-parietal functional connectivity reported in Bayraktaroğlu et al.^55^ could be attributed to the PD condition itself rather than being an effect of the MCI condition. This point can be the subject of further investigation.

### Differentiating PAC frequencies

The t-test hypothesis testing on the PAC features indicated highest values of − *log* (*p*-value) between PD groups in both drug conditions and the CTL group in the frequency ranges of 5–8 Hz for the phase frequency and 33–36 Hz for the amplitude frequency across all EEG channels. As a result, this frequency range was chosen for further investigation of the temporal dynamics of the PAC. This frequency range was reported in Axmacher et al.^41^ for the PAC activity during a working memory task. Furthermore, Roux and Uhlhaas^62^ demonstrated that the theta-gamma PAC in the medial and frontal areas plays a crucial role in coding sequential information in working memory. In the oddball task analyzed in the current study, working memory is involved as the subjects are instructed to count the target stimuli. In addition, the selected frequency range is a subset of the frequency range in which the PAC activity was observed during an attention task^38^. These studies provide more justifications for the suitability of the frequency range selected in this study.

### PAC dynamics and binding of sensory and memory data

The computational model of the hippocampal, basal ganglia, and frontal cortex circuits described in Atallah et al.^11^ (see Fig. 1) posits that a high-level path from the hippocampus to the frontal cortex goes through the ventral basal ganglia, and more specifically, the ventral striatum. There is also a high-level connection from the posterior cortex to the frontal cortex via the dorsal basal ganglia and the dorsal striatum. This computational model is based on the premise that producing the desired behavior, whether it is a physical response in the motor cortex or making a decision in the frontal cortex, is the consequence of binding two types of information in a specific manner. The first type is the learned or existing knowledge contributed by the hippocampus, and the second is the current sensory information reported through the posterior cortex. According to this computational model, the basal ganglia, and particularly its striatal regions, are in charge of establishing the desired data flow between the sources of these two types of information for the intended behavior to be produced. This notion is consistent with the activation of a gating mechanism by the basal ganglia which enables or disables an action in the motor cortex^27,63^ or in the prefrontal cortex such as updating the working memory^64,65^. Furthermore, the hippocampus plays an important role in the interaction of basal ganglia, particularly the ventral striatum, with the frontal cortex by modifying the state of bistable ventral striatum neurons^15,34^. Likewise, impacted by hippocampal inputs, midbrain dopaminergic neurons play an important role in the striatal neural state switching and, as a result, information processing in these regions^35^. Moreover, the dynamical activity of the striatum plays a critical role in learning stimulus–response behaviors or skill acquisition^13,14^. In addition to its gating effects on the working memory, the basal ganglia also serves a modulating role in fronto-posterior connectivity to bias the sensory information processing in favor of task-relevant information and thus control the attention mechanism^66^.

In the oddball task, subjects are asked to count the target stimuli. To do so, for each stimulus, the subject needs to make a decision on whether the current stimulus is the target or not, and then update the working memory in case of a target stimulus. As a result of the interaction between the hippocampus and the frontal cortex during working memory and goal-directed tasks^15,34,35,67,68^, it is expected that perceiving the target stimuli activates information flow from the hippocampus to the frontal cortex, potentially through the ventral striatum, and leads to forming a counting decision in the frontal cortex.

On the other hand, for the novelty stimuli, due to the presence of more complex patterns than in the target stimuli, a higher level of sensory information processing activity occurs. Given the role of the posterior cortex in sensory data representation^69^ and the basal ganglia’s influence on the fronto-posterior cortex to regulate the sensory processing and attention mechanisms^66^, it is expected that the novelty stimuli trigger the activity of the path from the posterior cortex to the frontal cortex which goes through the dorsal striatum. Consistent with this argument and the computational model of Atallah et al.^11^, the topography of the PAC for novelty stimuli in the two time intervals of 200–400 ms and 400–600 ms shown in Fig. 3c indicates considerable values for the PAC in posterior regions compared to that of the target stimuli (Fig. 3a).

For the target stimuli, however, the pattern of the PAC is concentrated on the centro-frontal regions and differs between the PD groups and the CTL group in terms of its temporal dynamics. These differences in PAC values for the target stimuli, which trigger the decision-making and working memory circuits in the brain, may be attributed to information flow via the high-level links between the hippocampus and frontal cortex via the ventral striatum (see Fig. 1), the most involved circuitry in PD.

For the novelty stimuli, due to its complicated sensory content, significant PAC values are present in both the posterior and frontal regions for the time interval of 200–400 ms (Fig. 3c). Given the differences between the CTL and PD groups in the PAC patterns for the novelty stimuli in posterior areas, the high PAC values can be linked to the connection between the posterior and frontal cortices via the dorsal striatum (see Fig. 1).

‌By way of conclusion, because of the impaired dopaminergic cells projecting to the striatum in PD patients and the significant increase in the dopamine level in the striatum compared to other regions after Levodopa use (i.e. for the PD ON group)^70^, these changes in spatial patterns of the PAC are likely attributable to the functionality of striatal circuits.

### Dopamine overload

Parkinson's disease is caused by the loss of dopaminergic cells in the SNc. Since this region has more connections with the dorsal striatum than the ventral striatum^71^, the level of dopamine in the dorsal striatum is more impacted by PD compared to the ventral striatum. Based on this argument, Cools^4^ proposed that Levodopa injection causes a dopamine overload in the ventral striatum. Given the importance of the hippocampal-frontal interaction for the target stimuli (mentioned above) and the path between the hippocampus and the frontal cortex through the ventral striatum (Fig. 1), the delay in the PAC peak for the target stimuli on channel Fz in Fig. 4b,d (and the cross-PAC peaks on Fz-F3, Fz-F4, Fz-FC3, Fz-FC4 in Fig. S1 in Supplementary Material), which only occurs for the PD ON group, can be associated with this overdose impairment in the ventral striatum caused by the administration of Levodopa. This delay for the PD ON group is also observed statistically in the histograms shown in Fig. S6 in Supplementary Material.

Furthermore, it is known that dopamine plays a significant role in reward prediction. Therefore, we can expect that Parkinson and dopamine-related medicine would affect this critical process in the brain. In this regard, Aarts et al.^88^ demonstrated that medication-related impairment in reward prediction performance is associated with impairment in the ventral striatum. This association is also consistent with the relevance of the midbrain dopaminergic-ventral striatal interaction in building a reward representation depending on the relevant stimulus information as predictive coding of the reward^17^. Consistent with these arguments and the important role of the hippocampus in the flow of information to the frontal cortex, our findings provide a possible explanation for Levodopa-induced impaired reward prediction performance as a result of delayed information flow to the frontal cortex via impaired circuits controlled by midbrain dopaminergic cells and the hippocampus^15,34,35^. Given the computational perspective presented in Atallah et al.^11^, this can also be viewed as delayed binding of information between the memory and sensory data by the hippocampus in associating reward to the current stimulus to form a desired action in the frontal cortex via the ventral striatum connection. This delayed information binding which manifests in late peaks in the Fz PAC and Fz-F3 cross-PAC for the PD ON group can thus explain impaired reward prediction performance by PD patients on Levodopa treatment.

### Reaction delay

A delay in commencing a motor response at the presentation of visual input has been reported as one of the cognitive impairments in Parkinson's disease^72^. As suggested in Stelmach et al.^72^, decomposing the reaction time to a stimulus into the interval between the stimulus onset and the electromyography (EMG) onset (premotor time), and the interval between the EMG onset and response initiation (motor time), led to the observation that the premotor time accounts for the initiation delay and is not associated with motor execution. Furthermore, the study attributed irregularities in the motor performance of PD patients to the failure of the basal ganglia to construct the necessary temporal dynamics of the motor program. Furthermore, Wang et al.^73^ and Philipova et al.^74^ discovered that PD patients showed a delayed reaction time to target stimuli in oddball tasks, which was associated with a delay in the occurrence of the N200 and P300 components in the ERP response.

According to our findings, this reaction delay or motor initiation delay could be attributed to delayed sensory information transfer from the posterior cortex to the frontal / motor cortices via the dorsal striatum, manifesting itself in the delay of the PAC peak on channel Pz shown in Fig. 4c,e (and of the cross-PAC peak on Pz-F3, Pz-F4, Pz-FC3, and Pz-FC4 shown in Fig. S2 in Supplementary Material). In addition, for both the PD ON and PD OFF groups, this delay is also observed statistically in the PAC peak interval histograms of Fig. S7 in Supplementary Material.

Furthermore, our findings provide further evidence for early information-processing abnormalities in PD patients performing an auditory oddball task^75^. The delay in the cross-PAC peak between channel Pz and frontal channels in both PD groups, and not just the PD OFF medication group, raises the possibility that this impairment is related to the impaired dynamics of dopamine release, not just the reduced dopamine level, in the dorsal striatum. This hypothesis is also consistent with the reported importance of creating a dynamic balance between flexibility and stability through different ranges of cognitive tasks by disseminating distinct levels of dopamine in different brain regions^76^.

### Habituation with PD and Levodopa

Another documented cognitive impairment in PD is linked to the so-called habituation process^77^. Intuitively, habituation is a form of the learning process, in which the information learned from previous experiences is valued more than the current sensory input, and as a result, the level of attention to the current stimuli is reduced. In terms of the computational model of information flow, when habituation occurs the information sent from the hippocampus to the frontal cortex (the learned information) contributes more to decision-making than the information sent from the posterior cortex to the frontal cortex (the sensory information). Consistent with this argument, the peaks of the Fz PAC and the Fz-F3 cross-PAC occur earlier for the second data block (Figs. 5a and 6a) (as well as the cross-PAC peaks for the Fz-F4, Fz-FC3, and Fz-FC4 pairs in Figs. S3-S5(a) in Supplementary Material). Thus, from the standpoint of information binding, the contribution of the learned information from the hippocampus results in an earlier process of binding for making a choice in the frontal cortex. It is noteworthy that the peak of the PAC at Pz and the cross-PAC for the Pz-F3 pair do not occur earlier in the second data block (Figs. 5b and 6b) (as well as the cross-PAC peaks involving Pz in Figs. S3-S5(b) in Supplementary Material), as sensory data processing and transfer are not affected by the learning and habituation processes. The early-occurrence of the frontal PAC peak in the second data block for the CTL group is also observed statistically in the histograms of Figs. S8-S12(a) and the lack of such advancement in the posterior PAC peaks can be seen in the histograms of Figs. S8-S12(b) in Supplementary Material.

Furthermore, such an advancement of the PAC peak in the frontal region in the second data block also occurs in the PD OFF group, albeit both the first and second block peaks occur one time interval later than those of the CTL group (Figs. 5a and 6a). Also, the histograms in Figs. S8-S12(a) in Supplementary Material indicate this early occurrence statistically for the PD OFF group. In contrast with both the CTL and PD OFF groups, an advancement of the PAC peak occurrence does not occur in the PD ON group as illustrated in Figs. 5 and 6 (as well as in Figs. S3-S5 and in the histograms of Figs. S8-S12(a) in Supplementary Material). This difference can potentially be attributed to the dopamine overdose in the ventral striatum which forms a connection between the hippocampus and the frontal cortex (see Fig. 1). In other words, this observation points to the possibility that cognitive impairments in PD may not be attributed only to the reduced dopamine levels in the striatum, and may be the result of defective dynamics in dopamine release, which becomes more severe for the PD ON group as a result of dopamine overdose in the ventral striatum. The association of impairment in the dynamics of the cross-PAC between the Fz and frontal channels with the high-level connection between the hippocampus and the striatum, and the observed impairment in habituation as a learning process, is consistent with observations reported in Tort et al.^78^ showing the importance of the PAC dynamics between the striatum and the hippocampus using local field potential recordings during sequential behavior learning and decision making.

Furthermore, as suggested in Yin et al.^14^, temporal dynamics of neural activity throughout different regions of the striatum play an important role in the automation of learned skills. Therefore, the difference in the dynamics of the PAC between the PD ON group and the other two groups on the two blocks of oddball data could reflect the impairment of neural activity dynamics in the striatum caused by Levodopa use, which leads to a deficit in the habituation process. This observation corroborates earlier reports on the effects of proper dopamine-based signaling on the quality of cognitive functions^79–82^.

## Methods

### Dataset

The dataset utilized in this study is an open-access dataset that was originally compiled and released by Cavanagh et al.^53^. The methods of data acquisition and a thorough explanation of the experimental tasks are provided in Cavanagh et al.^53^, and briefly described here.

Twenty-five (N = 25) individuals with PD and an equal number of matched controls of similar sex and age (CTL group) participated in the experiment. The task was a 3-way auditory oddball task. Three tones, including standard tones with a 440 Hz sinusoidal frequency (70% of trials), target tones with a 660 Hz sinusoidal frequency (15% of trials), and novelty distractors (15% of trials) were played for the participants. All sounds were presented for 200 ms, and an inter-trial interval (ITI) was randomly selected from a uniform distribution between 500 to 1000 ms. For the standard and target stimuli, an additional 450 ms was added to the ITI, resulting in an ITI value ranging from 950 to 1450 ms. This adjustment was made to ensure that there was no overlap in the pre-stimulus window during the ERP analysis. All the single tone sounds were played at 80 dB. The novelty sounds, selected from a naturalistic sound dataset, were played at a mean of 65 dB with an inter-quartile range of [− 6.5 dB, 6.5 dB]. The participants were asked to count the number of target stimuli mentally. The task contained two blocks of 100 trials with similar sequences of stimuli. At the end of each block, the participants were asked to report the count of target stimuli.

During the task, EEG signals using the standard 10–20 system were recorded. The signals were sampled at a rate of 500 samples per second on 64 channels while CPz was the online reference and the ground was placed at AFz. Also, the vertical electrooculogram (VEOG) was recorded from bipolar auxiliary inputs.

### Preprocessing

The EEGLab toolbox was used to perform all EEG data processing using an automatic procedure. First, all the non-EEG channels, namely the EOG channels, were removed, resulting in the remaining 63 channels. For each channel, the mean of EEG data was subtracted to remove the direct current offset of the data. Then a bandpass filter was applied with a low cut-off frequency of 1 Hz and a high cut-off frequency of 150 Hz. Afterwards, a 60 Hz notch filter with a 0.2 Hz width was applied. A script was then used to remove EEG channels with any of the following attributes:

1. A flat period of at least 5 s,
2. Line noise to signal ratio (measured as the ratio of standard deviation to the grand mean of the channel) of 12 times or more,
3. A smaller correlation with nearby channels than 0.8.

The removed channels were interpolated using the spherical method^83^. After the interpolation, all EEG channels, including the interpolated channels, were re-referenced to the average of all channels. The Artifact Subspace Reconstruction (ASR) algorithm^84^ was applied to remove artifacts from EEG signals. The maximum standard deviation for the removal of bad time windows by the ASR algorithm in EEGLab was set to 20 based on the visual check of preprocessed data for a number of sessions. Finally, the channels were referenced to the average EEG channel signal again in order to reset the EEG data to zero-sum across channels. All the above steps were also visually validated on the data of several participants to ensure the performance of the preprocessing steps. The mentioned steps were derived from Makato's preprocessing pipeline^85^.

After preprocessing, each channel’s signal was normalized to have a zero mean and a standard deviation of one. Then, to extract epochs for further analysis, the response to each stimulus was extracted from the recorded signal between − 200 ms before the stimulus onset and 1000 ms after the onset. Given the three types of stimuli in the sequence of the oddball task, the extracted epochs were clustered into three sets of standard, novelty, and target epochs. The number of epochs in the standard, novelty, and target clusters were 70, 15, and 15, respectively for each block of the task. Finally, the ERP signal was obtained for each of the stimuli types by averaging the epochs in the corresponding cluster.

### Phase-amplitude coupling

One of the problematic aspects of investigating the temporal dynamics of the PAC is the relationship of the time and frequency resolutions. The effect of a narrow bandpass filter limits the frequency resolution in traditional methods of calculation^86^. It is also necessary to study the fluctuations of the PAC in short time intervals, resulting in poor frequency domain resolution. A technique for addressing both of these issues was introduced in Munia and Aviyente^87^ and called Time Frequency Mean Vector Length (TF-MVL). We employed this method for calculating the PAC in our analysis. EEG signals are not considered as a Wide Sense Stationary (WSS) process in this technique. Thus, it is possible to distribute the energy of EEG signals over time and frequency. To do this, the Reduced Interference Rihaczek's (RID-Rihaczek) time–frequency distribution is used. The RID-Rihaczek distribution can be calculated using the following integral:

$$C\left( {t,f} \right) = \mathop \smallint \limits_{{}}^{{}} \mathop \smallint \limits_{{}}^{{}} exp\left( {\frac{{ - (\theta \tau )^{2} }}{\sigma }} \right)exp\left( {\frac{ - j\theta \tau }{\sigma }} \right)A\left( {\theta ,\tau } \right)exp\left( { - j\left( {\theta t + 2\pi f\tau } \right)} \right)d\tau d\theta$$

where $exp \left( {\frac{ - j\theta \tau }{\sigma }} \right)$ is the kernel function for the Rihaczek distribution, $exp \left( {\frac{{ - (\theta \tau )^{2} }}{\sigma }} \right)$ is the Choi-Williams kernel function used to filter cross-terms for multi-component signals, $A\left( {\theta ,\tau } \right)$ is calculated from the signal using the following formula:

$$A\left( {\theta ,\tau } \right) = \mathop \smallint \limits_{{}}^{{}} x\left( {u + \frac{\tau }{2}} \right)x^{*} \left( {u - \frac{\tau }{2}} \right)\left( {j\theta u} \right)du$$

and $x$ is the signal for which the PAC value is calculated. Having calculated the RID-Rihaczek distribution, the following formulas are used to calculate the amplitude of the high-frequency signal $A_{{f_{h} }} \left( t \right)$, and the phase activity of the low-frequency signal $\Phi_{{f_{l} }} \left( t \right)$:

$$A_{{f_{h} }} \left( t \right) = \mathop \smallint \limits_{{f_{a} }}^{{f_{b} }} C\left( {f,t} \right)df$$

$$\Phi_{{f_{l} }} \left( t \right) = arg \left[ {\frac{{C\left( {f_{l} ,t} \right)}}{{\left| {C\left( {f_{l} ,t} \right)} \right|}}} \right]$$

where $\left[ {f_{a} ,f_{b} } \right]$ is a small range around the target high-frequency $f_{h}$. Finally, the PAC is calculated using the Mean Vector Length (MVL) formula:

$$MVL\left( {f_{h} , f_{l} } \right) = \frac{1}{N}\mathop \sum \limits_{i = 1}^{N} A_{{f_{h} }} \left( {t_{i} } \right) exp \left( {j\Phi_{{f_{l} }} \left( {t_{i} } \right)} \right)$$

In this paper, all PAC values were calculated using the mentioned approach.

### PAC features

To investigate the dynamics of the PAC in different subject groups, the ERP signals were partitioned into 200 ms windows, resulting in 6 windows for each ERP signal. For calculating the PAC, the phase frequency and the amplitude frequency were swept from 1 to 20 Hz and from 1 to 80 Hz, respectively, with a step size of 1 Hz on both ranges. For the given frequency ranges, we calculated the PAC for each ERP signal. Since there were three groups of subjects and three types of stimuli, nine types of ERP signals and, correspondingly, nine types of PAC values were produced for each channel. Therefore, nine PAC distributions for the mentioned frequency ranges were calculated for each channel. Partitioning each PAC distribution into frequency bins of 4 Hz on each frequency axis, the average of the PAC values within each bin was calculated to create a new feature matrix for each PAC distribution. This is equivalent to lowpass filtering the 2D PAC distribution using a $4\times 4$ uniform window and downsampling by a factor of 4 (see Fig. S13 in Supplementary Material).

Based on partitioning the ERP signal into 6 time intervals, a vector of 6 PAC feature values in the selected frequency bin was produced for each ERP signal. Then, taking the grand mean for these vectors of PAC feature values for all subjects within each group, nine PAC grand mean vectors were obtained corresponding to the collection of subject groups and stimulus types.

The t-test hypothesis testing was applied to the new PAC feature values over all ERP intervals for the target stimulus of each channel and each frequency bin between all pairs of subject groups, and the log *p*-value was aggregated over all channels. Then, the Benjamini–Hochberg correction was applied to the aggregated *p*-values to identify the most discriminating frequency regions separating the CTL group from the PD groups in both medication conditions. After selecting the target frequency region, the PAC values of different channels and cross-channels were examined to find the most distinguishing channels between the CTL and PD groups across all grand mean vectors. As a result, channels Pz and Fz were selected, and their PAC values as well as the cross-channel PAC values between each of these two channels and channels FC3, FC4, F3, and F4 were used for further analysis.

### Cross-channel PAC time series

Selected out of 63 channels with high PAC values, channels Fz, Pz, F3, F4, FC3, and FC4 were used for applying PAC calculations between pairs of channels. In the cross-channel method of PAC calculation, the phase of the signal from one channel and the amplitude of the signal from another channel are used to calculate the mutual PAC associated with the pair of channels. The cross-channel PAC was calculated between Fz and Pz as the amplitude channels and F3, F4, FC3, and FC4 as the phase channels for each of the six time intervals during the ERP signal. Taking the grand mean of the PAC values in each case for each subject group, nine types of cross-channel PAC vectors were produced, each corresponding to a subject group and a stimulus type. These vectors were then compared to each other to identify meaningful differences between the dynamics of the PAC for different subject groups.

### Statistical analyses

T-tests were applied to all statistical comparisons and the Benjamini–Hochberg correction was applied to the aggregated *p*-values when multi-comparison tests were conducted. To compare the PAC values between different types of stimuli associated with each time interval and subject group, paired difference t-tests were used since fixing the time interval and subject group, the PAC samples are paired with each other. This is also the case for comparing PAC values between time intervals for each subject group to statistically show the existence of particular trends in the PAC dynamics. Moreover, paired t-tests were used for comparison between two PD groups, PD ON and OFF medication, since both groups consist of the same individuals going through different medication conditions. For comparison between each PD group and the CTL group, however, independent t-tests were used.

To examine statistical changes in the time interval of the PAC peak occurrence for each subject group or between the two data blocks for the same group, histograms of the PAC peak time interval indices were produced and the index of the time interval with highest probability was selected as the maximum likelihood estimation of the PAC peak time interval.

## Conclusion

Our results provide evidence for the hypothesis that the main manifests of cognitive impairment in PD are not merely attributable to the level of dopamine deficit but are a reflection of the temporal dynamics of dopamine-based signaling. Furthermore, we showed that the dynamics of the PAC in an oddball task can be used to assess the cognitive side effects of the standard pharmaceutical treatment method for PD based on Levodopa. Our findings demonstrate distinguishing patterns in the PAC dynamics between different subject groups and medication conditions, indicating that changes in temporal dynamics of dopamine release may contribute to cognitive impairments present in PD as well as those induced by Levodopa. Although the association of the PAC calculated from EEG data to the neural pathway activities through the striatum is not straightforward, the dynamical behavior of the PAC derived this way can help provide an explanation for the delay in reaction time and initiation of motor response and for the impaired habituation in PD patients, which is consistent with the computational models of the basal ganglia circuits and the role of high-level information gating mechanisms in cognitive processes. In addition, these findings can have important implications in developing appropriate mechanisms for drug administration such as technologies for time-controlled release of dopamine.

### Supplementary Information

Supplementary Information.

## Data Availability

The dataset analyzed in this study was produced by authors of an article published earlier and information about accessing the data can be found within that article: J. F. Cavanagh, P. Kumar, A. A. Mueller, S. P. Richardson, and A. Mueen, “Diminished EEG habituation to novel events effectively classifies Parkinson’s patients,” *Clin Neurophysiol*, vol. 129, no. 2, pp. 409–418, Feb. 2018, https://doi.org/10.1016/j.clinph.2017.11.023.

## Code availability

The code developed in this study can be found at: https://github.com/AIRLab-Sharif/Parkinson-PAC.
