# Supplementary material for: Aberrations in temporal dynamics of cognitive processing induced by Parkinson’s disease and Levodopa
Source: Sci Rep. 2023 Nov 18;13:20195. doi: 10.1038/s41598-023-47410-3 (PMC10657430; doi:10.1038/s41598-023-47410-3)
Supplement: Supplementary file 1 — Supplementary Information. [file 41598_2023_47410_MOESM1_ESM.docx]

Supplementary Material

Aberrations in temporal dynamics of cognitive processing

induced by Parkinson’s disease and Levodopa

**Mohammad Mahdi Kiani^1^, Mohammad Hossein Heidari Beni^1^, Hamid Aghajan^1*^**

^1^Department of Electrical Engineering, Sharif University of Technology, Tehran, Iran

***Correspondence:** [aghajan@ee.sharif.edu](mailto:aghajan@ee.sharif.edu)

## **PAC dynamics**

As discussed in the main text, there is a late-occurrence of the peak in the Fz PAC and Fz-F3 cross-channel PAC for the PD ON group, in marked contrast to the other subject groups. Similar late-occurrence of the cross-PAC peak can also be seen for Fz-F4, Fz-FC3 and Fz-FC4, as shown in Fig. S1.


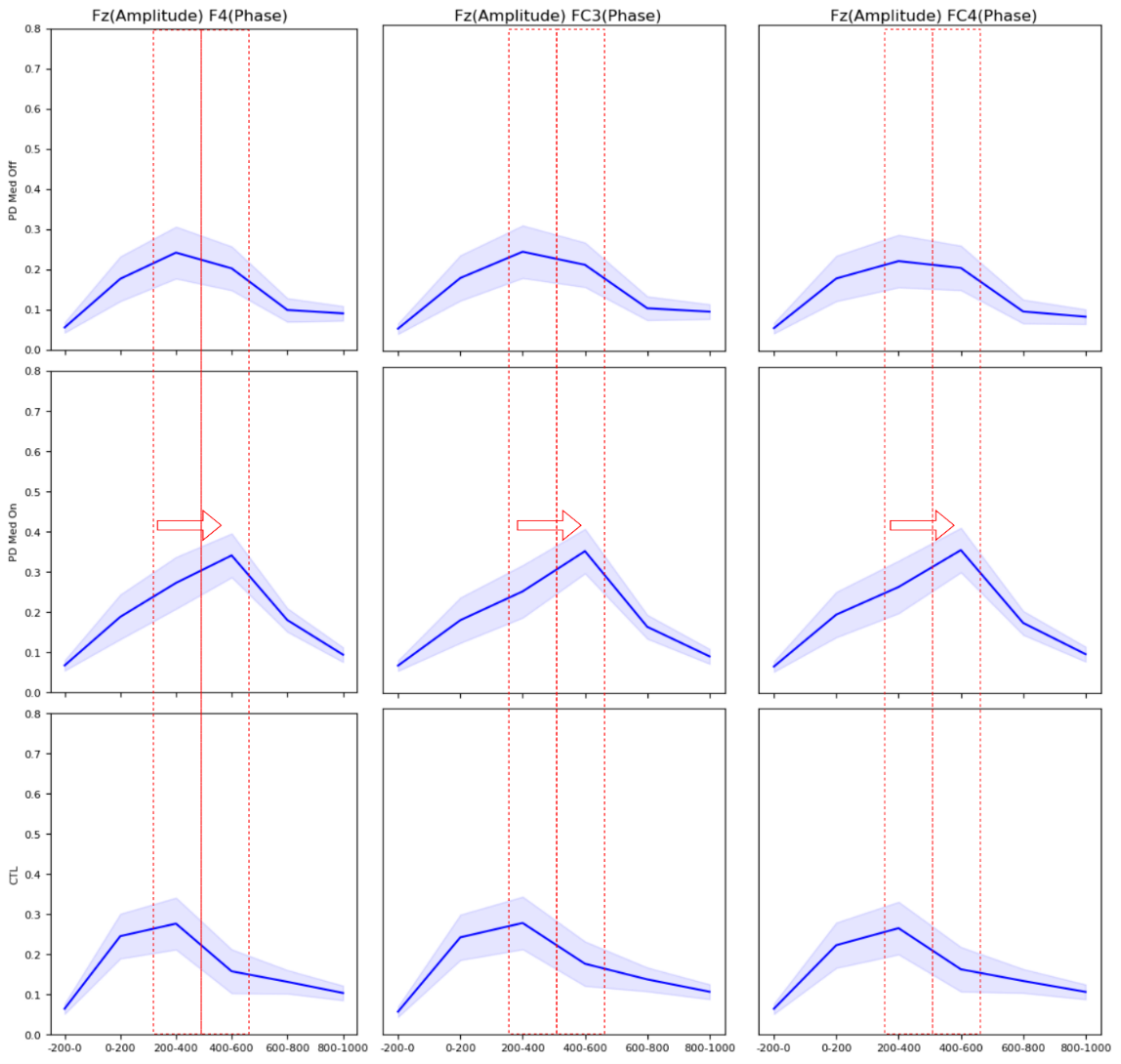


*Figure S1. Grand mean PAC dynamics for the target stimulus with 90% confidence interval in cross-channels Fz-F4, Fz-FC3, and Fz-FC4. As red arrows show, for the PD ON group, the increasing trend of the PAC continues for a longer period compared to the other two groups.*

In the case of channel Pz, the late-occurrence of the PAC peak and the Pz-F3 cross-PAC peak is observed for both PD groups (and not just the PD ON group). Similar late-occurrence of the cross-PAC peak can also be seen for both PD groups for Pz-F4, Pz-FC3, and Pz-FC4, as shown in Fig. S2.


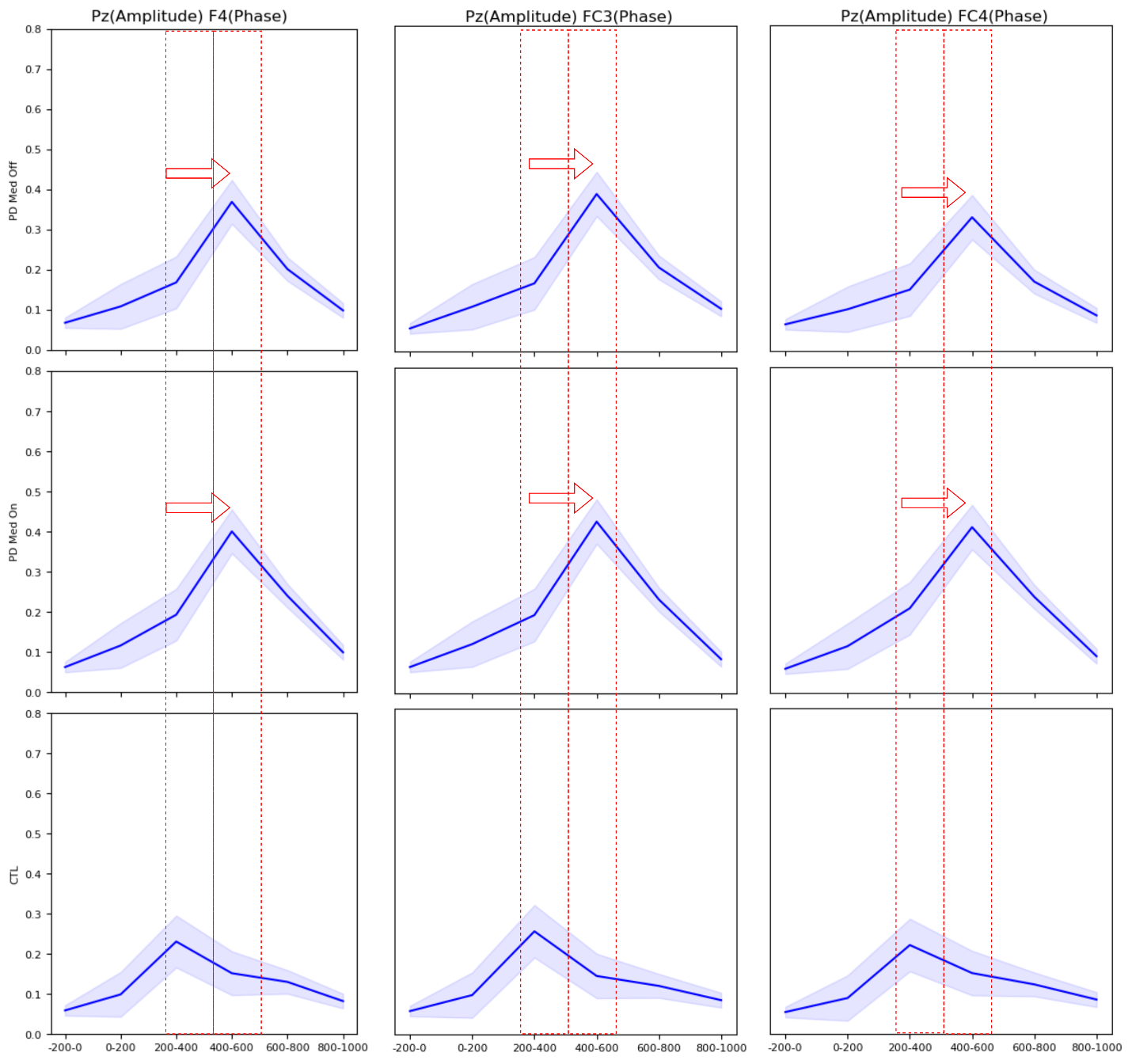


*Figure S2. Grand mean PAC dynamics for the target stimulus with 90% confidence interval in cross-channels Pz-F4, Pz-FC3, and Pz-FC4. As the red arrows show, for both PD groups, the increasing trend of the PAC continues for a longer period compared to the CTL group.*

**Comparing PAC dynamics between two block runs**

The early-occurrence of the PAC peak in the second block of the oddball task occurs for both the CTL and PD OFF groups in cross-channel PAC for Fz-F4, Fz-FC3, and Fz-FC4, as illustrated in Figs. S3-5(a). However, such early-occurrence of the PAC peak in the second data block cannot be seen for the Pz-F4, Pz-FC3, and Fz-FC4 cross-channel PAC as shown in Figs. S3-5(b). Furthermore, for both blocks of the oddball task, the PAC peak for both PD groups in cross-channels involving Pz also occurs later, in the time interval of 400-600 ms (see Figs. S3-5(b)). However, for the cross-channels involving Fz, the late-occurrence of the PAC peak is observed only for the PD ON group, as shown in Figs. S3-5(a).


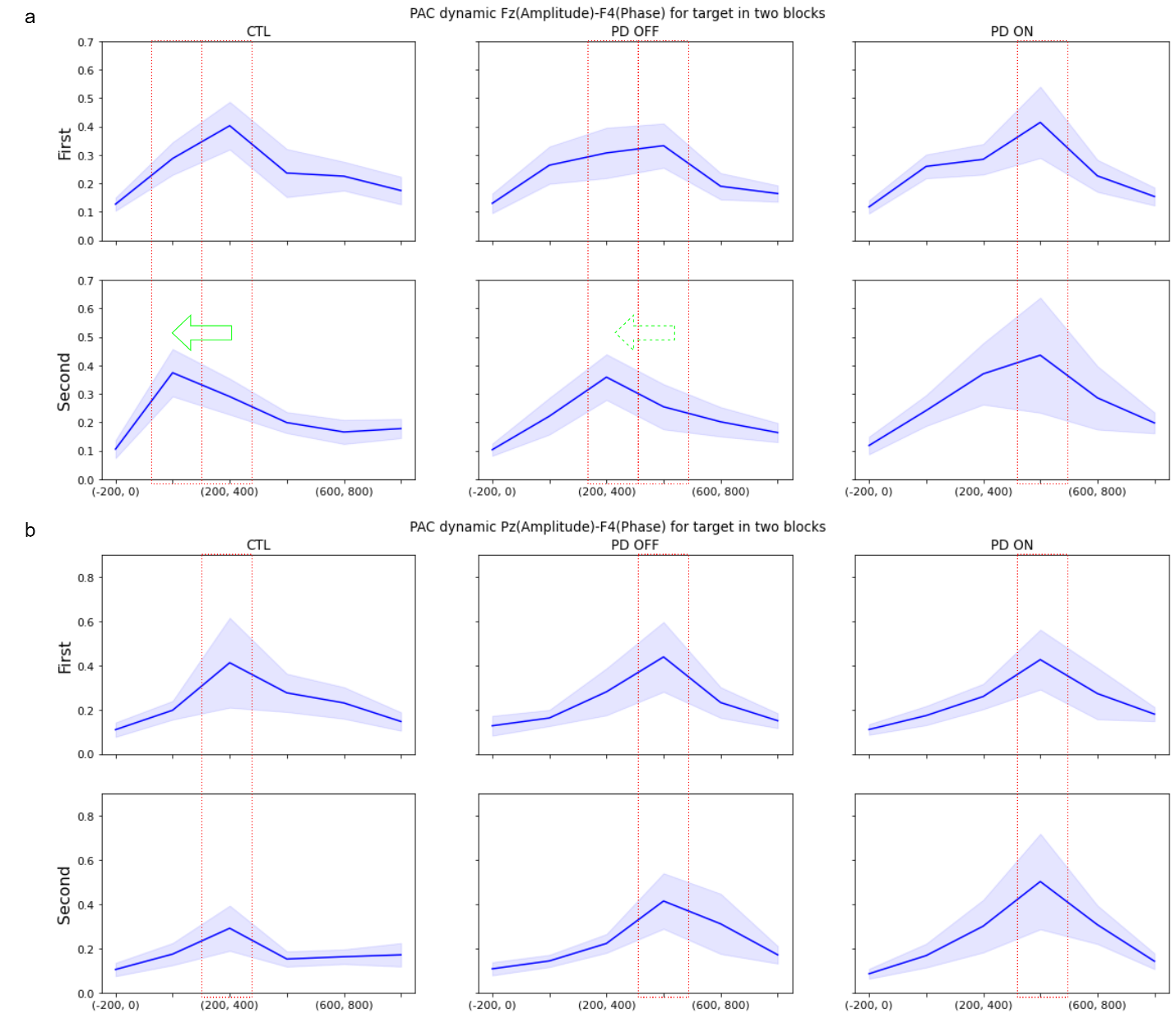


*Figure S3. Grand mean of the PAC dynamics for cross-channel PAC in a) Fz-F4 and b) Pz-F4 for the target stimulus in two blocks of the oddball task. The green arrow in part a shows the early-occurrence of the PAC peak in the second block for cross-channel PAC Fz-F4 in the CTL group. This early-occurrence of the PAC peak is also observed for the PD OFF group (indicated by the dotted green arrow); however, this early-occurrence of the PAC peak has a delay of one time interval compared to the CTL group. In marked contrast, there is no such early occurrence of the PAC peak for the PD ON group. In case of cross-channel Pz-F4, there is no early-occurrence of the PAC peak in the second block for any group. However, the grand mean of the cross PAC Pz-F4 shows a delay of one time interval in the occurrence of the peak for both PD groups compared to the CTL group for both data blocks.*


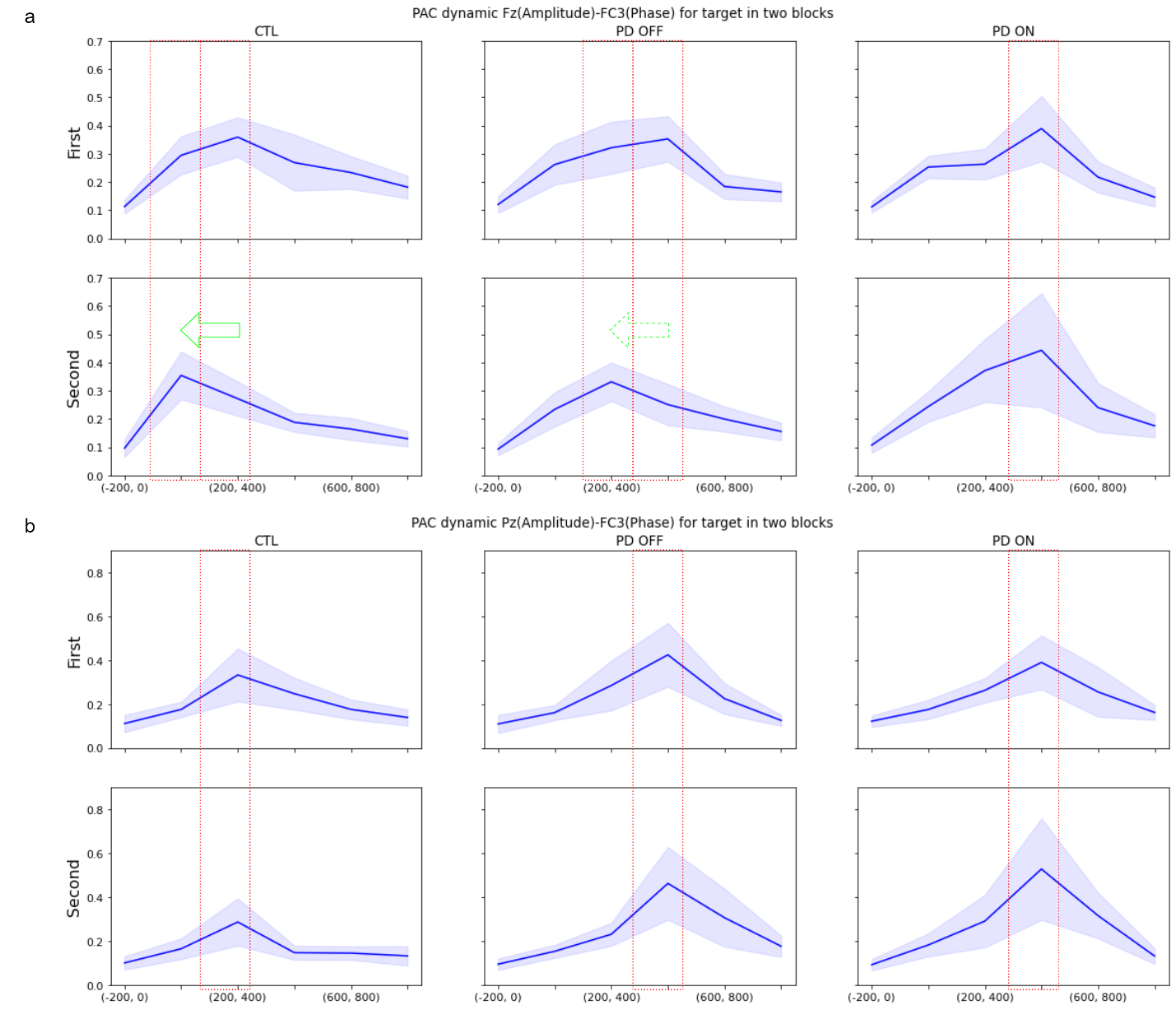


*Figure S4. Grand mean of the PAC dynamics for cross-channel PAC in a) Fz-FC3 and b) Pz-FC3 for the target stimulus in two blocks of the oddball task. The green arrow in part a shows the early-occurrence of the PAC peak in the second block for cross-channel PAC Fz-FC3 in the CTL group. This early-occurrence of the PAC peak is also observed for the PD OFF group (indicated by the dotted green arrow); however, this early-occurrence of the PAC peak has a delay of one time interval compared to the CTL group. In marked contrast, there is no such early occurrence of the PAC peak for the PD ON group. In case of cross-channel Pz-FC3, there is no early-occurrence of the PAC peak in the second block for any group. However, the grand mean of the cross PAC Pz-F4 shows a delay of one time interval in the occurrence of the peak for both PD groups compared to the CTL group for both data blocks.*


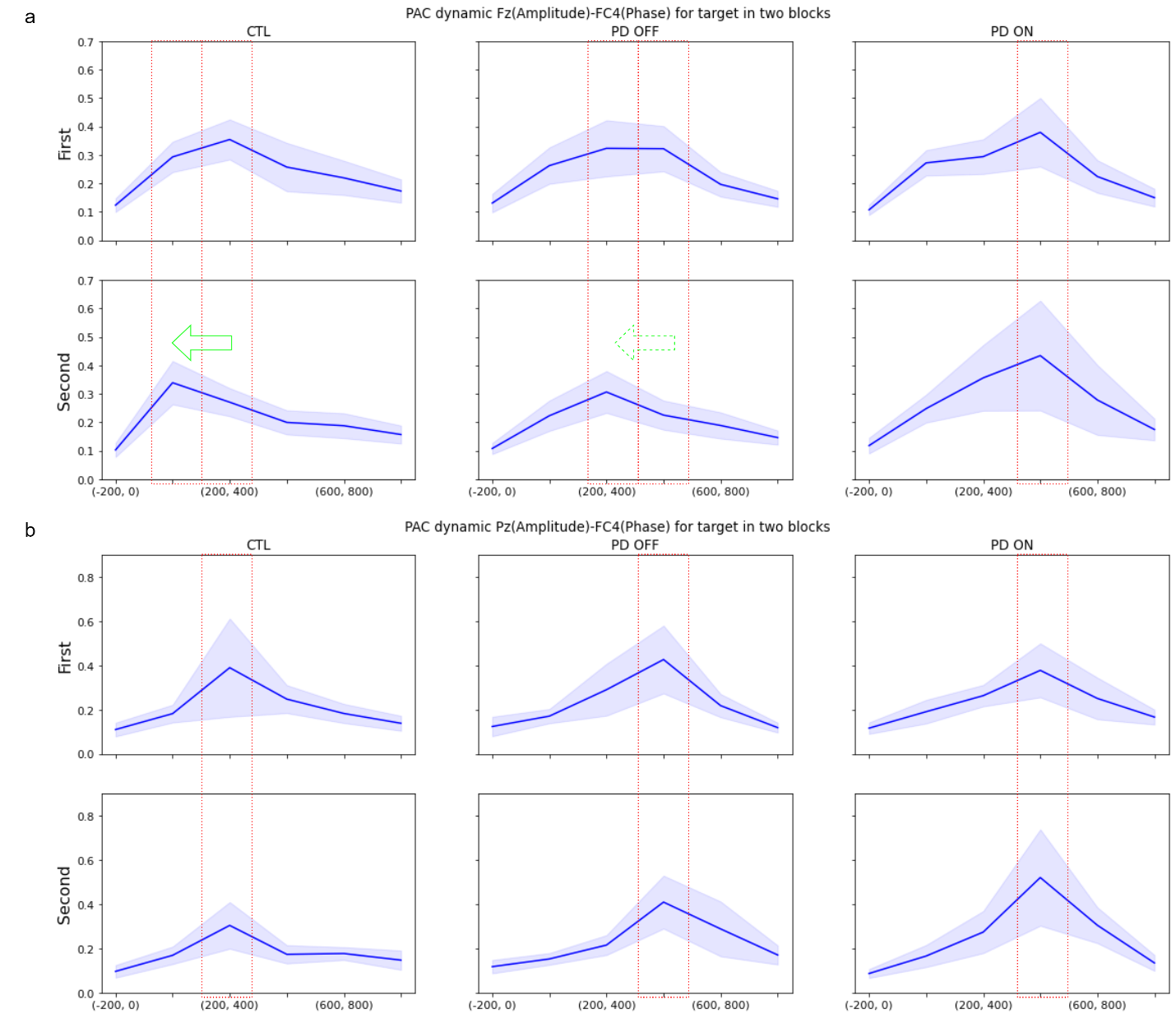


*Figure S5. Grand mean of the PAC dynamics for cross-channel PAC in a) Fz-FC4 and b) Pz-FC4 for the target stimulus in two blocks of the oddball task. The green arrow in part a shows the early-occurrence of the PAC peak in the second block for cross-channel PAC Fz-FC4 in the CTL group. This early-occurrence of the PAC peak is also observed for the PD OFF group (indicated by the dotted green arrow); however, this early-occurrence of the PAC peak has a delay of one time interval compared to the CTL group. In marked contrast, there is no such early occurrence of the PAC peak for the PD ON group. In case of cross-channel Pz-FC4, there is no early-occurrence of the PAC peak in the second block for any group. However, the grand mean of the cross PAC Pz-F4 shows a delay of one time interval in the occurrence of the peak for both PD groups compared to the CTL group for both data blocks.*

**Histograms of PAC dynamics**

The histograms of Fig. S6 reveal that it is more probable for the PD ON group to have the maximum PAC for channel Fz and cross-channels Fz-FC3, Fz-FC4, Fz-F3, and Fz-F4 at a later time than for the PD OFF and CTL groups. According to these histograms, the most probable time interval for the maximum PAC occurrence in the PD ON group is 400-600 ms, while the PAC peak for both the CTL and PD OFF groups occurs in the 200-400 ms interval. This observation is consistent with the delay observed in Figs. 1 and 4 of the main article and Fig. S1 for the maximum PAC on channel Fz and its related cross-channels for the PD ON group in comparison to the other two groups.

On the other hand, as the histograms shown in Fig. S7 suggest, it is more probable for both PD ON and PD OFF groups to have their PAC peak for Pz and its related cross-channels in the 400-600 ms interval while these peaks occur in the interval of 200-400 ms for the CTL group. This observation is also consistent with latencies of the PAC peaks for Pz and its cross-channels for all groups shown in Figs. 1(c, e) of the main article and Fig. S2.


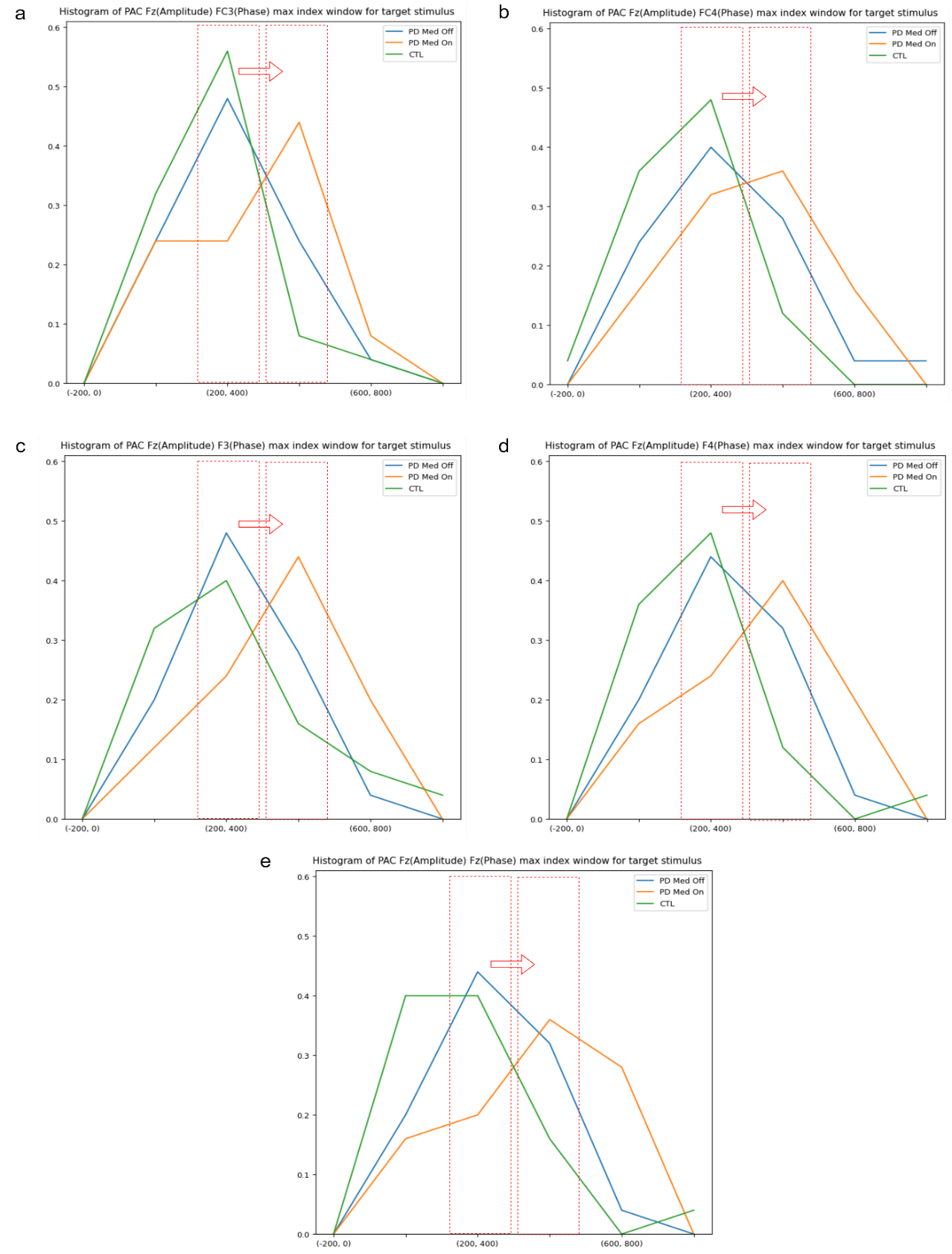


*Figure S6. Histograms of the PAC peak time intervals for a) Fz-FC3, b) Fz-FC4, c) Fz-F3, d) Fz-F4, and e) Fz-Fz. For the CTL group (green curves) and the PD OFF group (blue curves), the interval 200-400 ms is the most probable interval for the occurrence of the PAC peak, while for the PD ON group (orange curves), the most probable interval for the occurrence of the PAC peak is 400-600 ms.*

*Figure S7. Histograms of the PAC peak time intervals for a) Pz-FC3, b) Pz-FC4, c) Pz-F3, d) Pz-F4, and e) Pz-Pz. For the PD ON group (orange curves) and the PD OFF group (blue curves), the interval 400-600 ms is the most probable interval for the occurrence of the PAC peak, while for the CTL group (green curves), the most probable interval for the occurrence of the PAC peak is 200-400 ms.
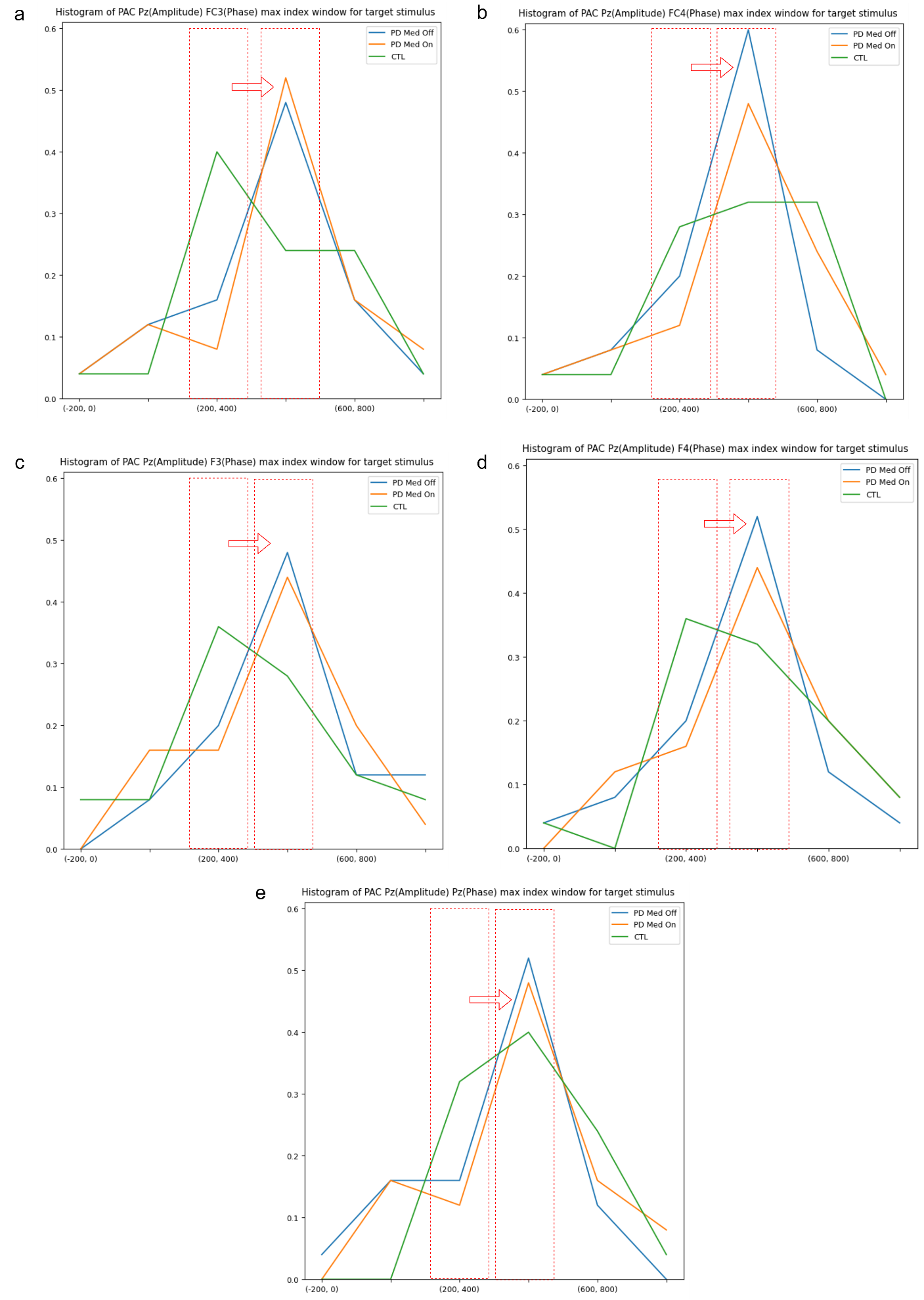
*

**Histograms of PAC dynamics between two block runs**

Starting with the CTL group for analyzing the dynamics of the PAC between the two experiment blocks, as part (a, left) of Figs. S8-12 show, in the first block, the interval of 200-400 ms is the most probable interval for the occurrence of maximum PAC in Fz and cross-channels associated with it. In the second block, however, the probability of the occurrence of maximum PAC for these channels increases for the 0-200 ms interval and matches or surpasses the level of the 200-400 ms interval.

For the PD OFF group, the maximum PAC occurs in the interval 400-600 ms for Fz and its cross-channels in the first block, and in the interval 200-400 ms in the second block (part (a, middle) of Figs. S8-12). This means that while the PAC peak occurs with one time interval delay in this group, similar to the CTL group, an advancement of the time of the PAC peak to an earlier interval is observed for this group in the second data block. In contrast with the other two subject groups, no significant change in the distribution of the maximum PAC interval is observed for the PD ON group for the mentioned channels (part (a, right) of Figs. S8-12).

For Pz and its related cross-channels, however, there is no significant difference between the distributions of the max PAC interval between the first and second blocks for all subject groups (see Figs. S8-12(b)). Furthermore, in both data blocks, the maximum PAC is more probable to occur in the 400-600 ms interval for both PD groups whereas is more likely to occur in the 200-400 ms interval for the CTL group.


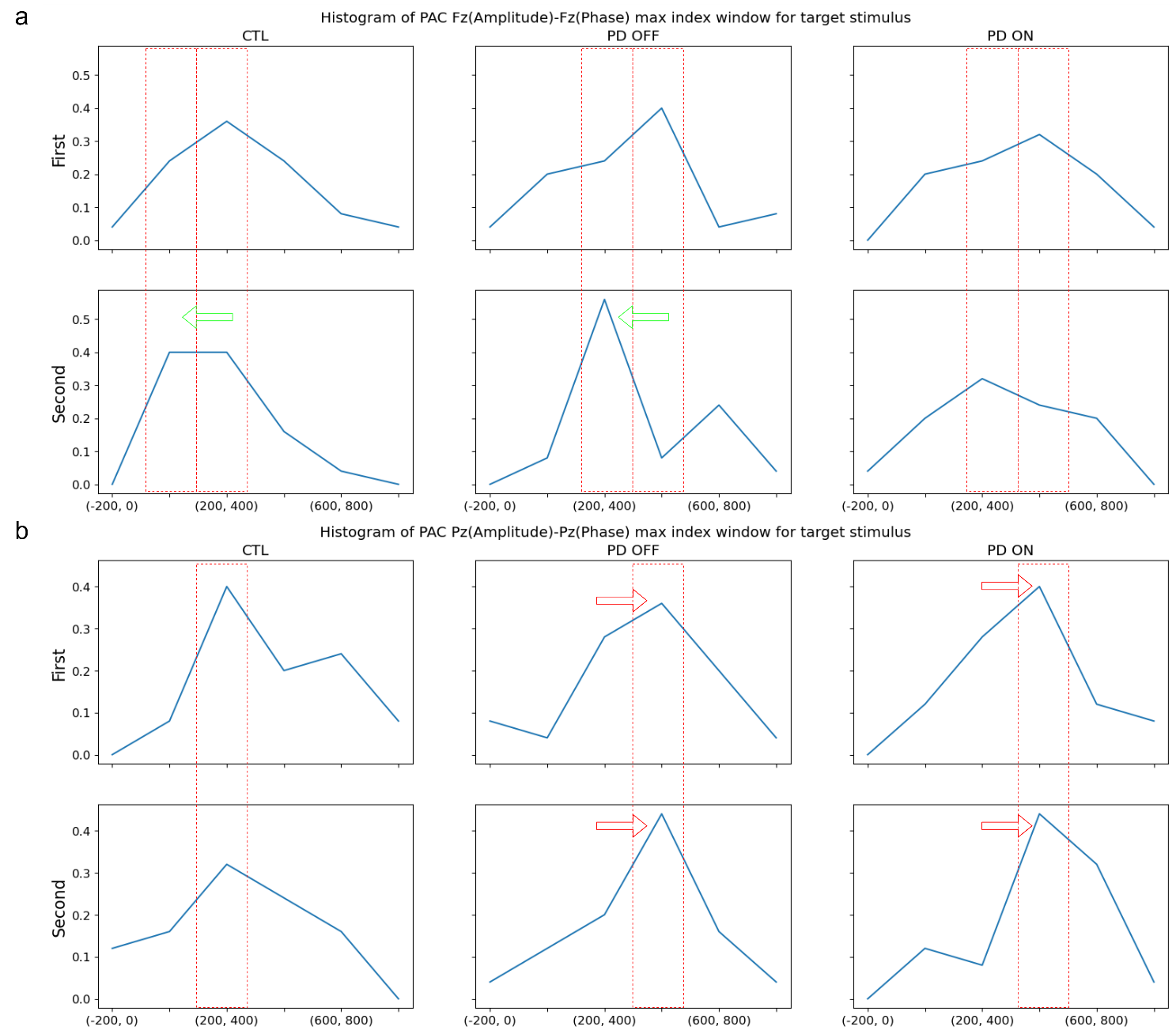


*Figure S8. Histograms of maximum PAC time intervals for channels: a) Fz and b) Pz. First rows indicate histograms of maximum PAC intervals in the first block of the oddball task for the three subject groups. The Green arrows show that the maximum probable time for the occurrence of the PAC peak for Fz moves to an earlier interval in the second block for the CTL and PD OFF groups. This shift for the PD OFF group is from the 400-600 ms interval to the 200-400 ms interval whereas for the CTL group the shift is from the 200-400 ms interval to the 0-200 ms interval. For the PD ON group, there is no significant shift in the maximum probable interval for Fz. For Pz, there is no clear change in the distribution of the maximum PAC intervals for any subject group. Furthermore, for both blocks, the most probable maximum PAC interval for both PD groups is 400-600 ms in marked contrast with that of the CTL group which is 200-400 ms.*


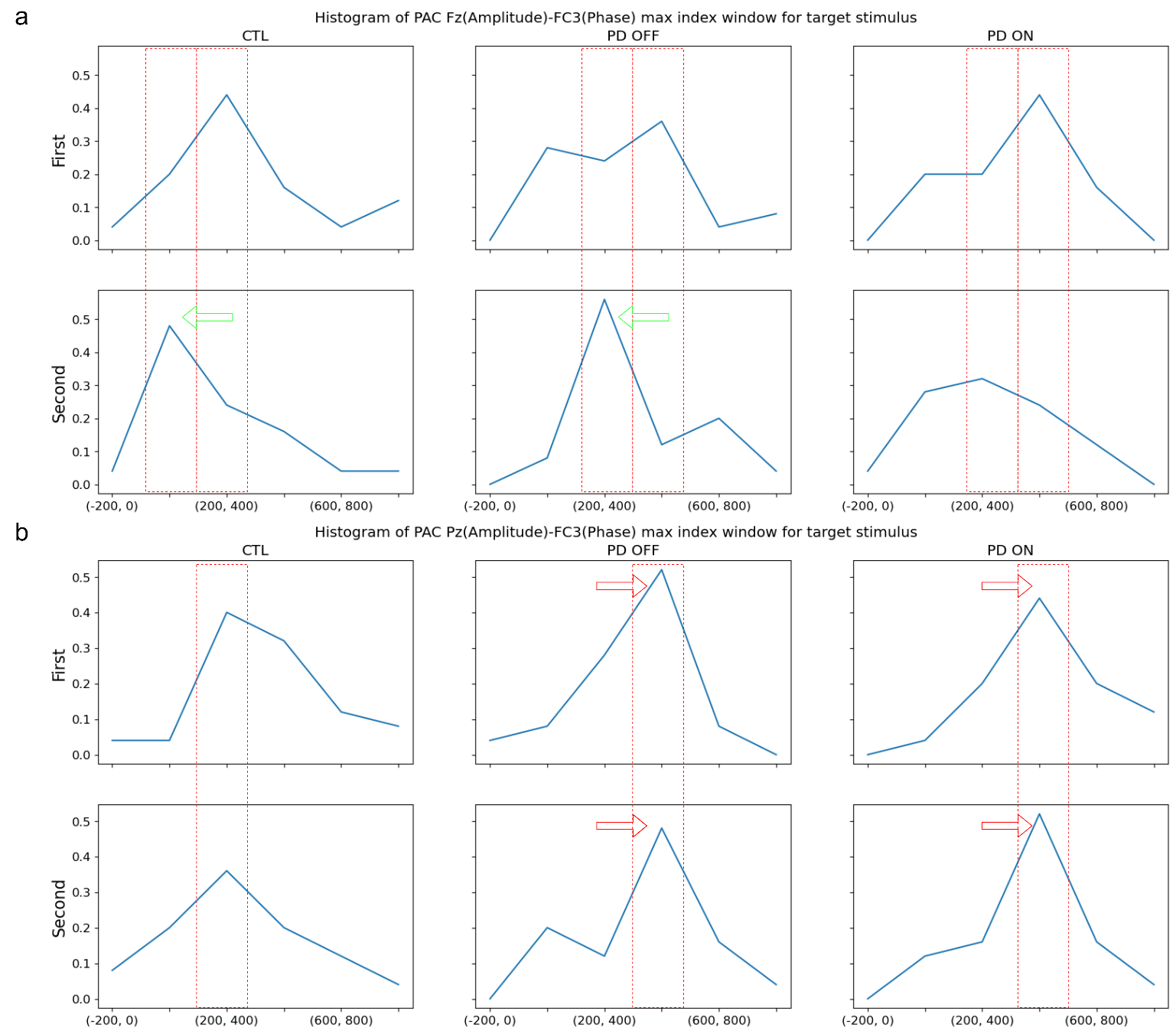


*Figure S9. Histograms of maximum PAC time intervals for cross-channels: a) Fz-FC3 and b) Pz-FC3. First rows indicate histograms of maximum PAC intervals in the first block of the oddball task for the three subject groups. The Green arrows show that the maximum probable time for the occurrence of the PAC peak for Fz-FC3 moves to an earlier interval in the second block for the CTL and PD OFF groups. This shift for the PD OFF group is from the 400-600 ms interval to the 200-400 ms interval whereas for the CTL group the shift is from the 200-400 ms interval to the 0-200 ms interval. For the PD ON group, there is no significant shift in the maximum probable interval for Fz-FC3. For Pz-FC3, there is no clear change in the distribution of the maximum PAC intervals for any subject group. Furthermore, for both blocks, the most probable maximum PAC interval for both PD groups is 400-600 ms in marked contrast with that of the CTL group which is 200-400 ms.*


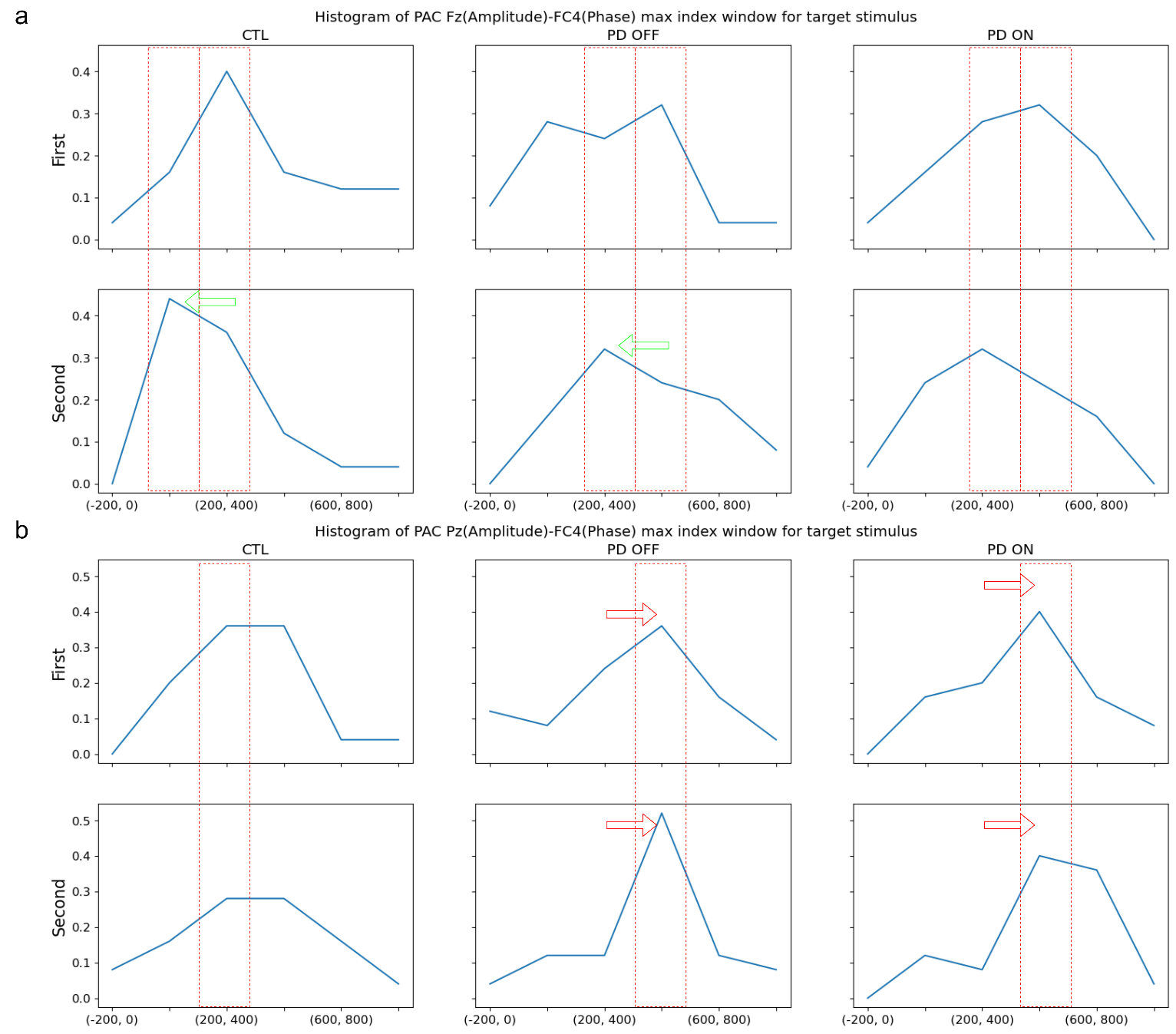


*Figure S10. Histograms of maximum PAC time intervals for cross-channels: a) Fz-FC4 and b) Pz-FC4. First rows indicate histograms of maximum PAC intervals in the first block of the oddball task for the three subject groups. The Green arrows show that the maximum probable time for the occurrence of the PAC peak for Fz-FC4 moves to an earlier interval in the second block for the CTL and PD OFF groups. This shift for the PD OFF group is from the 400-600 ms interval to the 200-400 ms interval whereas for the CTL group the shift is from the 200-400 ms interval to the 0-200 ms interval. For the PD ON group, there is no significant shift in the maximum probable interval for Fz-FC4. For Pz-FC4, there is no clear change in the distribution of the maximum PAC intervals for any subject group. Furthermore, for both blocks, the most probable maximum PAC interval for both PD groups is 400-600 ms in marked contrast with that of the CTL group which is 200-400 ms.*


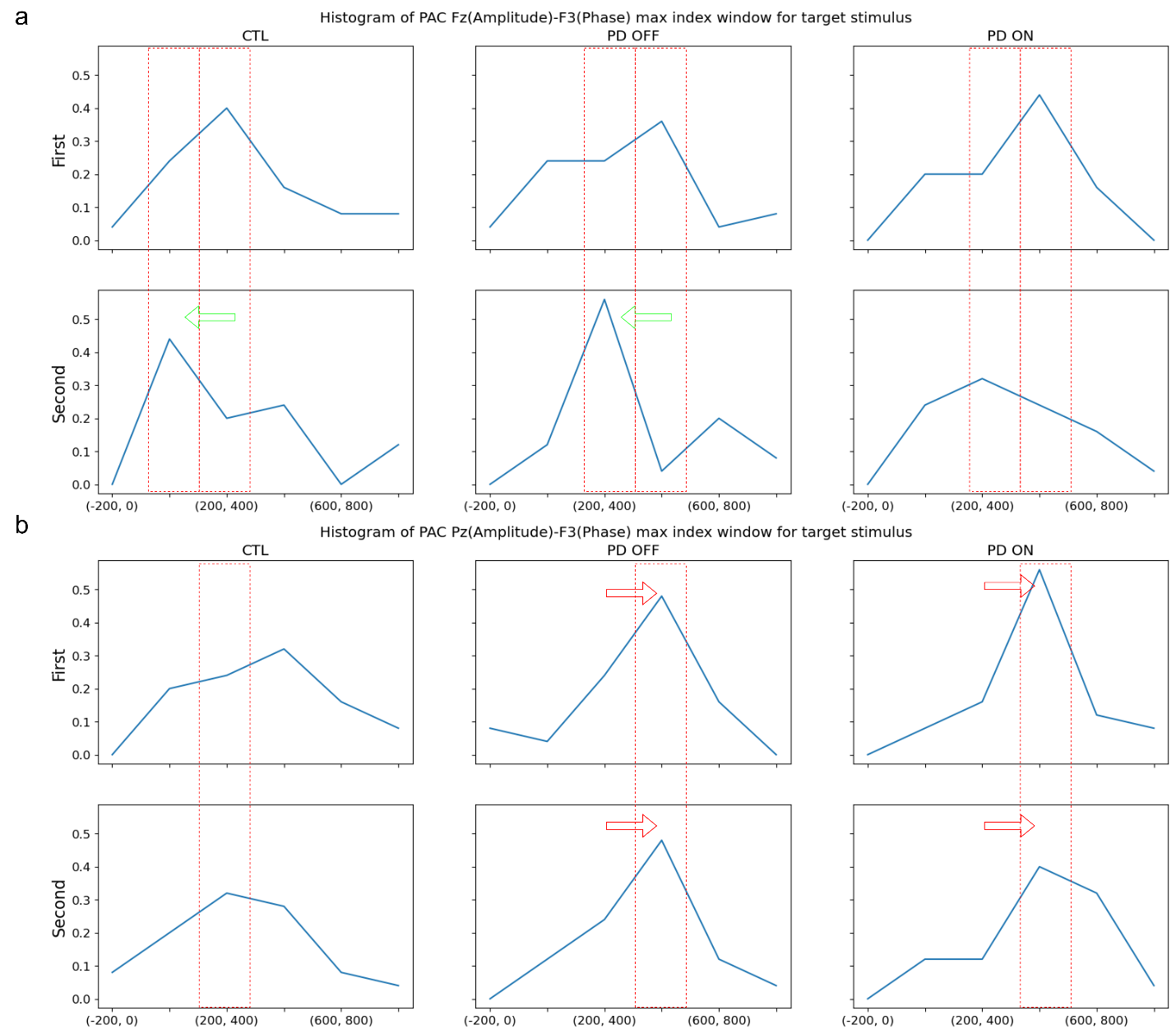


*Figure S11. Histograms of maximum PAC time intervals for cross-channels: a) Fz-F3 and b) Pz-F3. First rows indicate histograms of maximum PAC interval in the first block of the oddball task for the three subject groups. The Green arrows show that the maximum probable time for the occurrence of the PAC peak for Fz-F3 moves to an earlier interval in the second block for the CTL and PD OFF groups. This shift for the PD OFF group is from the 400-600 ms interval to the 200-400 ms interval whereas for the CTL group the shift is from the 200-400 ms interval to the 0-200 ms interval. For the PD ON group, there is no significant shift in the maximum probable interval for Fz-F3. For Pz-F3, there is no clear change in the distribution of the maximum PAC intervals for any subject group. Furthermore, for both blocks, the most probable maximum PAC interval for both PD groups is 400-600 ms in marked contrast with that of the CTL group which is 200-400 ms.*


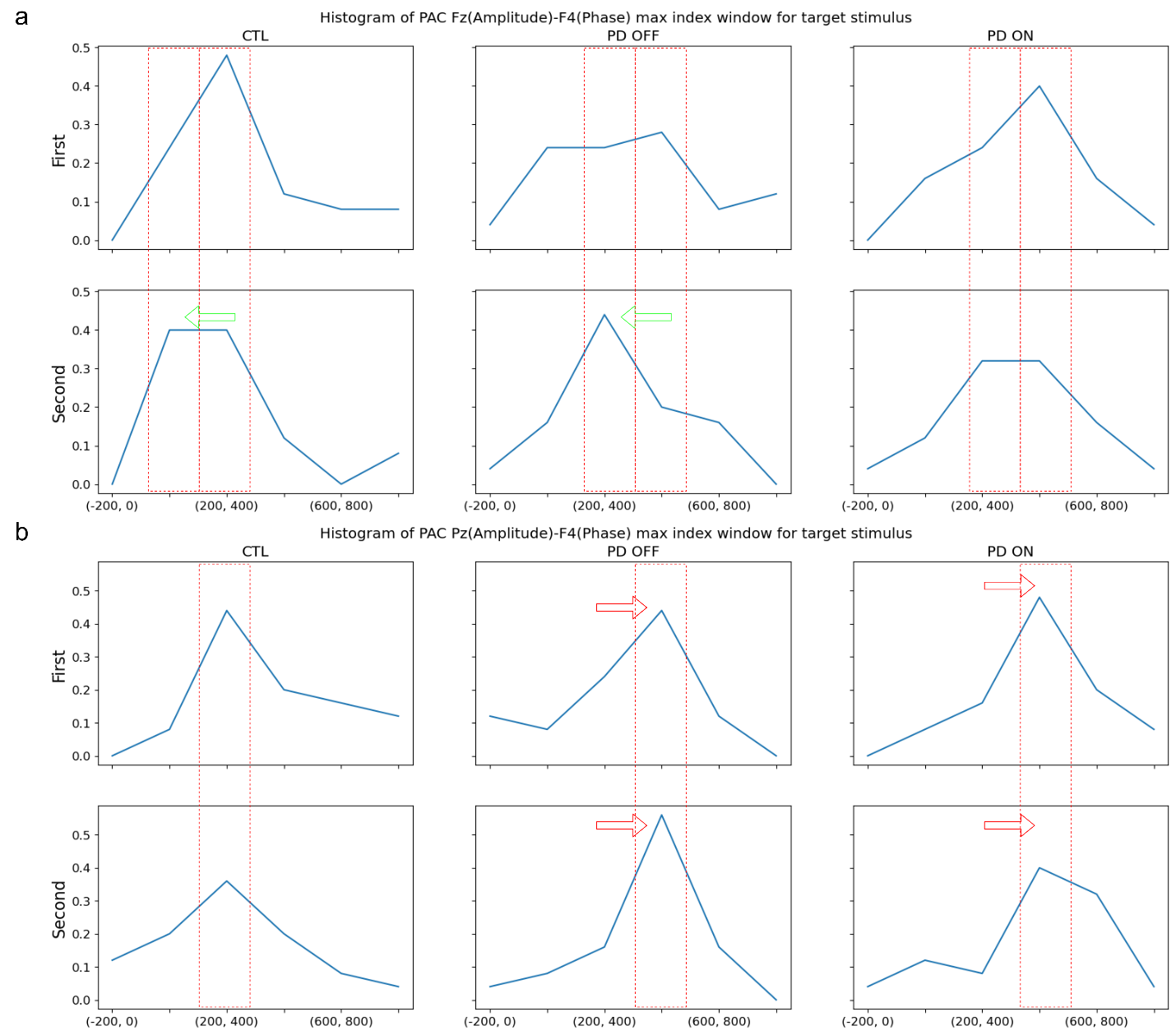


*Figure S12. Histograms of maximum PAC time intervals for cross-channels: a) Fz-F4 and b) Pz-F4. First rows indicate histograms of maximum PAC interval in the first block of the oddball task for the three subject groups. The Green arrows show that the maximum probable time for the occurrence of the PAC peak for Fz-F4 moves to an earlier interval in the second block for the CTL and PD OFF groups. This shift for the PD OFF group is from the 400-600 ms interval to the 200-400 ms interval whereas for the CTL group the shift is from the 200-400 ms interval to the 0-200 ms interval. For the PD ON group, there is no significant shift in the maximum probable interval for Fz-F4. For Pz-F4, there is no clear change in the distribution of the maximum PAC intervals for any subject group. Furthermore, for both blocks, the most probable maximum PAC interval for both PD groups is 400-600 ms in marked contrast with that of the CTL group which is 200-400 ms.*

**Downsampling method on PAC features**

The original PAC matrix containing PAC values for each pair of low and high frequencies at a resolution of 1 Hz was downsampled as depicted in Fig. S13. As can be observed, the average of the PAC values was calculated for a frequency region with a width and height of 4Hz. The resulting value formed a component of the new matrix presented in Fig. S13, termed the PAC feature matrix.


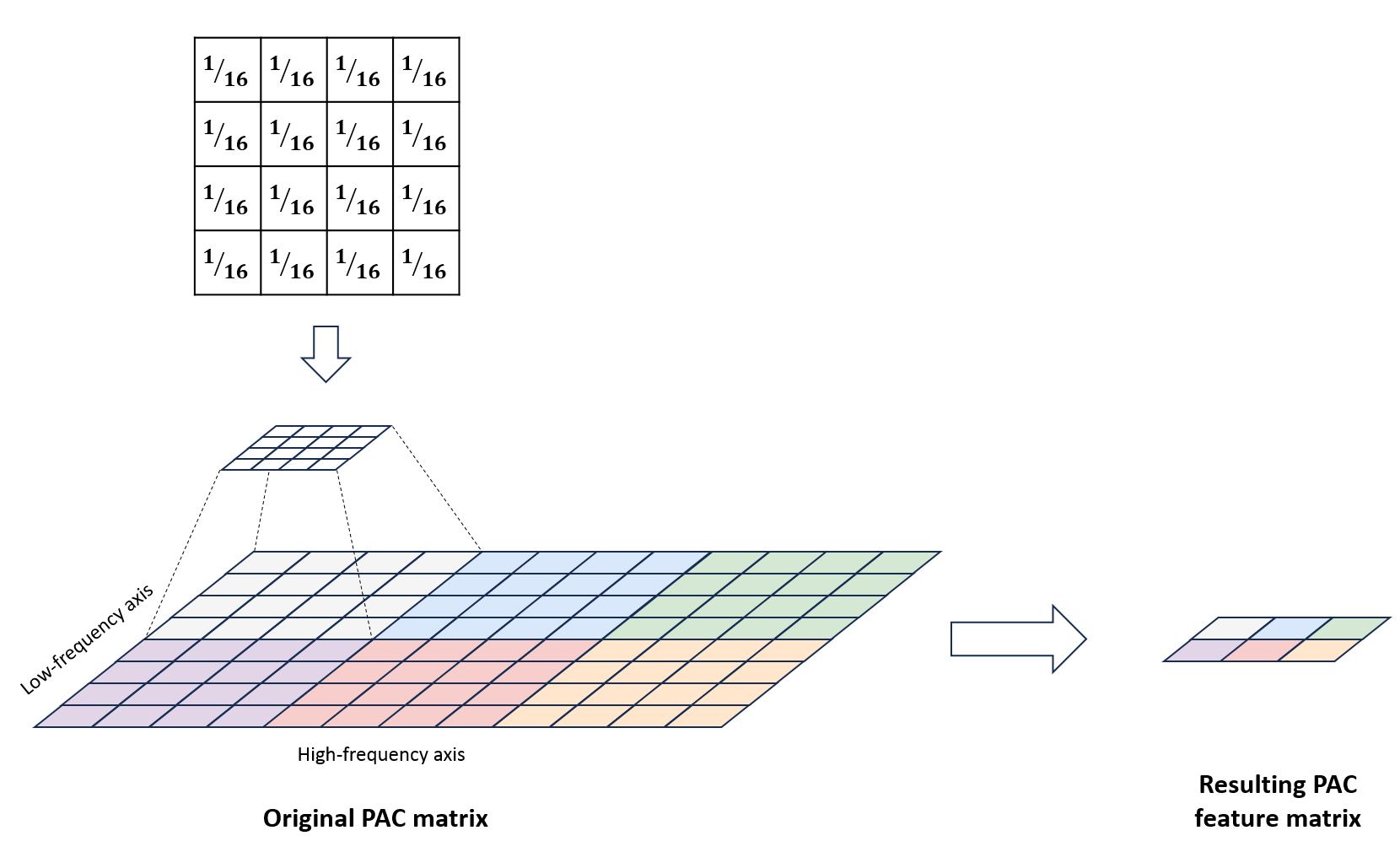


Figure S13. The process of downsampling of the PAC matrix.
